# Supplementary material for: mixOmics: An R package for ‘omics feature selection and multiple data integration
Source: PLoS Comput Biol. 2017 Nov 3;13(11):e1005752. doi: 10.1371/journal.pcbi.1005752 (PMC5687754; doi:10.1371/journal.pcbi.1005752)
Supplement: S1 Text — (PDF) [file pcbi.1005752.s001.pdf]

S1 Supplementary Information
mixOmics: an R package for ‘omics feature selection and multiple data integration

Florian Rohart [1,5], Benoît Gautier [1], Amrit Singh [2,3], K-A. Lê Cao [1,4*]

[1]The University of Queensland Diamantina Institute,
The University of Queensland, Translational Research Institute, QLD 4102, Australia,
[2]UBC James Hogg Research Centre for Heart Lung Innovation, St. Paul’s Hospital and
[3]Prevention of Organ Failure (PROOF) Centre of Excellence, Vancouver, BC, Canada
[4]Centre for Systems Genomics and School of Mathematics and Statistics, University of Melbourne, VIC 3010, Australia
[5]Current address: Institute for Molecular Bioscience, The University of Queensland, Brisbane, QLD 4072, Australia

* kimanh.lecao@unimelb.edu.au

Contents

1 General definitions 3
1.1 General notations . . . 3
1.2 Cross-validation . . . 3
1.3 Prediction distances . . . 3

2 Graphical outputs to visualise multivariate analysis results 4
2.1 Sample plots . . . 5
2.2 Variable plots . . . 6
2.3 Additional graphical outputs . . . 7

3 Single ‘omics supervised multivariate analysis with PLS-DA and sPLS-DA 7
3.1 PLS - Discriminant Analysis . . . 7
3.2 Variable selection with sparse PLS-DA . . . 8
3.3 Extensions of PLS-DA for repeated measurements and 16S microbiome data . . . 9
3.4 Workflow for PLS-DA analysis . . . 9

4 N-integration across multiple ‘omics data sets with DIABLO 9
4.1 DIABLO method . . . 10
4.2 Specific outputs to visualise multiple ‘omics data sets integration . . . 10
4.3 Workflow for DIABLO analysis . . . 11

5 P-integration across independent data sets with MINT 11
5.1 MINT method . . . 11
5.2 Leave-One-Group-Out Cross-Validation for performance assessment . . . 12
5.3 Specific graphical outputs for MINT . . . 12
5.4 Workflow for MINT analysis . . . 12

6 Computational time for large data sets 12

List of Figures

1 **Example of sample plot with `plotIndiv` and loading plot with `plotLoadings` with sPLS-DA**. The loading plot (right) represents the top 80 genes selected on the second component of the sPLS-DA model. Colors indicate the tumour subtype where the mean expression levels of the gene is maximal. More details in the 'Results' Section R and sweave code 3.4. 7
2 **Summary of the main `mixOmics` S3 functions for supervised multivariate analyses.** 8
3 **Example of data matrix decomposition for single 'omics analysis with PLS-DA.** The predictor matrix X is decomposed into a set of components $(t_1, \ldots, t_H)$ and associated loading vectors $(a_1, \ldots, a_H)$, $\boldsymbol{Y}$ is the outcome coded as a dummy indicator matrix as described in Table 1 and combined linearly (see exact formula in Equation (2)). $\boldsymbol{X_h}$ is the deflated (residual) matrix starting with $\boldsymbol{X_1} = \boldsymbol{X}$, for $h = 1 \ldots H$. H is the dimension of the model - or the number of components. 9
4 **Example of different design matrices in `DIABLO` for the multi-'omics breast cancer study** (illustrated in the main article). Links or cells in grey are added by default in the `DIABLO` function `block.plsda` and `block.splsda` and do not need to be specified by the user. 11

List of Tables

1 Example of an input outcome factor y (left) transformed into an indicator dummy matrix Y (right). The transformation is performed internally in the supervised multivariate methods. 3
2 Example of predicted dummy variables $\widehat{Y}_{\text{new}}$ corresponding to each class, and predicted class based on maximum distance on test samples from the SRBCT data. The true class of the test samples is indicated in the row names. 5
3 Example of centroids coordinates for each class from the learning set (left table), predicted scores and predicted class based on the centroid distances for 3 components (right table) on test samples from the SRBCT data. The true class of the test samples is indicated in the row names. 5
4 Example of runtime for very large data sets analysed in `mixOmics`. Tuning and performance assessments were performed with 5-fold CV for single 'omics and N-integration, or LOGOCV for P-integration (Rohart et al. 2017, Singh et al. 2016, cluster with 10 cpus and 50 Gb RAM). 13

1. General definitions

1.1. General notations

We assume each data set has been normalised using appropriate techniques specific for the type of 'omics platform prior to the `mixOmics` analysis. Let X denote a data matrix of size N observations (rows) $\times$ P predictors (e.g. expression levels of P genes, in columns). The categorical outcome y is expressed as a dummy indicator matrix Y where each column represents one outcome category and each row indicates the class membership of each sample. Thus, Y is of size N observations (rows) $\times$ K categories outcome (columns). Table 1 illustrates the transformation from a categorical outcome y into an indicator dummy matrix Y (right). The transformation is performed internally in `mixOmics`. We denote for all $a \in \mathbb{R}^n$ its ℓ^1 norm $||a||_1 = \sum_1^p |a_j|$ and its ℓ^2 norm $||a||_2 = (\sum_1^p a_j^2)^{1/2}$. For any matrix we denote by $^\top$ its transpose. In the methods discussed in this article, the data X are internally centered and scaled, as proposed by default in PLS-based methods (Wold, 1975).

Table 1: Example of an input outcome factor y (left) transformed into an indicator dummy matrix Y (right). The transformation is performed internally in the supervised multivariate methods.

	y
indiv1	trt1
indiv2	trt1
indiv3	trt1
indiv4	trt1
indiv5	trt2
indiv6	trt2
indiv7	trt2
indiv8	trt2
indiv9	trt3
indiv10	trt3
indiv11	trt3
indiv12	trt3

	trt1	trt2	trt3
indiv1	1	0	0
indiv2	1	0	0
indiv3	1	0	0
indiv4	1	0	0
indiv5	0	1	0
indiv6	0	1	0
indiv7	0	1	0
indiv8	0	1	0
indiv9	0	0	1
indiv10	0	0	1
indiv11	0	0	1
indiv12	0	0	1

1.2. Cross-validation

Cross-validation (CV) is a model validation technique used in statistical and machine learning to assess whether the results of an analysis can be generalised to an independent data set. It consists in dividing the data set into s subsets (or folds), fitting the model on $s-1$ subsets and evaluating the prediction performance on the left-out subset. This process is iterated until each subset is left out once; the prediction performance are then averaged. In our methods, prediction performance refers to either an overall misclassification error rate or a balanced error rate calculated on the left-out samples.

We define *stratified CV* when there is approximately the same proportion of each class in each of the folds. Repeated cross-validation implies that the whole CV process is repeated a number of times `nrepeat` to reduce variability across the different subset partitions. In the case of Leave-One-Out CV (`validation = 'loo'`), each sample is left out once ($s = N$) and therefore `nrepeat` is set to 1.

For the P- integration with `MINT`, we use *Leave-One-Group-Out CV* as described in Section 5.2.

1.3. Prediction distances

As mentioned in the main article, different prediction distances are proposed and implemented in the functions `predict`, `tune` and `perf` to assign to each new observation a final predicted class.

Mathematically, we can define those predicted outputs for a model with H components as follows. Recall that the outcome matrix Y is a dummy matrix of size $N \times K$. For N_{new} new observations and their

expression matrix X_{new}, we define the *predicted dummy variables* $\widehat{Y}_{\text{new}}$ of size $N_{\text{new}} \times K$ as:

$$\widehat{Y}_{\text{new}} = X_{\text{new}} * W(D^\top W)^{-1}B$$

where W, D and B are derived from the X and Y training data sets. W is a $P \times H$ matrix containing the loading vectors associated to X, D is a $P \times H$ matrix containing the regression coefficients of X on its H latent components and B is a $H \times K$ matrix containing the regression coefficients of Y on the H latent components associated to X. Therefore, $\widehat{Y}_{\text{new}}$ is the prediction from a multivariable (several columns) multivariate model.

We define the *predicted scores* or *predicted latent variables* (components) T_{pred} of size $N_{\text{new}} \times H$ as:

$$T_{\text{pred}} = X_{\text{new}} * W(D^\top W)^{-1}$$

with the same notations as above. The prediction distances are then applied as follows:

- The maximum distance `"max.dist"` is applied to the predicted dummy values $\widehat{Y}_{\text{new}}$ and is the most intuitive method to predict the class of a new observation sample. The predicted class is the outcome category with the largest predicted dummy value. The distance performs well in single data set analysis with multiclass problems Lê Cao et al. (2011).

For the centroid-based distances 'Mahalanobis distance' and 'Centroids distance', we first calculate the centroid G_k of all the learning set samples belonging to the class $k \leq K$ based on the H latent components associated to X. Both 'Mahalanobis distance' and 'Centroids distance' distances are applied on the predicted scores T_{pred}. The predicted class of a new observation is

$$\underset{1 \leq k \leq K}{\operatorname{argmin}} \left\{ \operatorname{dist}(T_{\text{pred}}, G_k) \right\}, \quad (1)$$

i.e the class for which the distance between its centroid and the H predicted scores is minimal, for a given distance defined as:

- The centroid distance `"centroids.dist"` solves (1) using the Euclidian distance $\operatorname{dist}(x, G_k) = \sqrt{\sum_{h=1}^{H} (x_h - (G_k)_h)^2}$
- The Mahalanobis distance `"mahalanobis.dist"` solves (1) using the Mahalanobis distance $\operatorname{dist}(x, G_k) = \sqrt{(x - G_k)^\top S^{-1}(x - G_k)}$, where S is the variance-covariance matrix of $x - G_k$.

In practice we found that the centroid-based distances, and specifically the Mahalanobis distance led to more accurate predictions than the maximum distance for complex classification problems and N-integration problems. The centroid distances consider the prediction in a H dimensional space using the predicted scores, while the maximum distance considers a single point estimate using the predicted dummy variables on the last dimension of the model. The user can assess the different distances and choose the prediction distance that achieves the best performance using the `tune` and `perf` outputs (see workflows 3.4, 4.3 and 5.4).

We output an example of the predicted dummy variables $\widehat{Y}_{\text{new}}$ (Table 2), predicted coordinates T_{pred} (Table 3) and associated distance predictions for a PLS-DA analysis on the SRBCT data set (analysed from the data in worflow 3.4).

2. Graphical outputs to visualise multivariate analysis results

`mixOmics` (Lê Cao et al., 2017) aims to provide insightful and user-friendly graphical outputs to interpret statistical and biological results, some of which (correlation circle plots, relevance networks and clustered image maps) were introduced in González et al. (2012). Via the use of R/S3 functions listed in 2, the function calls are identical for all multivariate methods implemented in the `mixOmics` package, as we illustrated in the Result section. We offer various visualisations, including sample plots and feature plots, which are based on the component scores and the loading vectors, respectively. Here we list the main important visualisation functions in `mixOmics` for which outputs are shown in Figure 1 of the manuscript.

Table 2: Example of predicted dummy variables $\widehat{Y}_{\text{new}}$ corresponding to each class, and predicted class based on maximum distance on test samples from the SRBCT data. The true class of the test samples is indicated in the row names.

	EWS	BL	NB	RMS	max.dist
EWS.T6	0.77	-0.17	0.00	0.39	EWS
EWS.T7	0.89	-0.11	-0.04	0.26	EWS
EWS.T14	0.89	-0.01	-0.11	0.23	EWS
EWS.C11	0.96	-0.10	0.27	-0.13	EWS
NB.C2	0.35	0.01	0.53	0.11	NB
NB.C9	0.11	-0.04	0.92	0.01	NB
NB.C8	0.08	0.04	0.82	0.06	NB
RMS.C9	0.24	-0.08	0.22	0.62	RMS
RMS.C5	-0.15	0.14	0.40	0.60	RMS
RMS.T4	0.24	0.06	-0.06	0.76	RMS
RMS.T5	0.23	0.05	0.03	0.70	RMS
RMS.T3	0.42	0.05	-0.11	0.64	RMS
RMS.T11	0.19	0.10	-0.08	0.79	RMS

Table 3: Example of centroids coordinates for each class from the learning set (left table), predicted scores and predicted class based on the centroid distances for 3 components (right table) on test samples from the SRBCT data. The true class of the test samples is indicated in the row names.

	dim 1	dim 2	dim 3
EWS	-8.79	-11.83	-3.61
BL	26.34	-6.19	12.47
NB	7.83	10.87	-16.36
RMS	-8.16	12.61	8.29

	dim.1	dim.2	dim.3	centroid.dist	mahalanobis.dist
EWS.T6	-17.52	-3.56	0.45	EWS	EWS
EWS.T7	-16.13	-7.99	0.13	EWS	EWS
EWS.T14	-13.85	-9.91	2.46	EWS	EWS
EWS.C11	-6.83	-10.77	-11.74	EWS	EWS
NB.C2	3.37	3.41	-11.66	NB	NB
NB.C9	11.16	10.46	-21.47	NB	NB
NB.C8	11.43	9.83	-17.51	NB	NB
RMS.C9	-9.14	8.98	0.71	RMS	NB
RMS.C5	4.00	14.58	0.12	RMS	RMS
RMS.T4	-10.91	6.34	10.40	RMS	RMS
RMS.T5	-9.14	6.88	7.55	RMS	RMS
RMS.T3	-12.35	1.82	9.38	RMS	RMS
RMS.T11	-9.93	6.83	11.94	RMS	RMS

2.1. Sample plots

Sample plots display the component scores, and therefore visualises similarities between samples in a reduced dimensional space spanned by the first few latent components of the model.

`plotIndiv`. For the integrative methods (e.g. `DIABLO` and `MINT`), samples from separate data set are plotted on separate figures, allowing to assess the agreement between the data sets at the sample level. Confidence ellipse plots for each class can be displayed. To visualise the prediction areas of each class, users can overlay prediction results to sample plots via the `background` input parameter (see 'Prediction area visualisation' Section and Figure 2 in the main article). The method define surfaces around samples that belong to the same predicted class. These surfaces are then be used to shade the background of the sample plot. More details are provided in our help file `?background.predict`. This functionality is currently only implemented for supervised analyses of single 'omics analysis (`plsda, splsda`) with no more than 2 dimensions.

`plotArrow` (Arrow representation). The plot overlays the components scores from multiple datasets and draws arrows between scores associated to the same sample. For most supervised methods and two-'omics integration methods listed in Table 1, the start of the arrow represents the component score in the X

data set and the tip of the arrow is the component score associated to the outcome, or the Y data set. In particular, for N-integration, the start of the arrow indicates the centroid between all ‘omics data sets for a given sample and the tips of the arrows the location of the same sample in each data set. In two-‘omics and N-integration methods, short arrows indicate a strong agreement between the matching data sets, long arrows a disagreement between the matching data sets (see Figure 3 in main article).

2.2. Variable plots

Variable plots aim to display the correlations between selected variables across data sets, by using the latent components as a surrogate variable to estimate the correlations (correlation circle plots) or associations between variables (clustered image maps and relevance networks). The loading vectors plot displays the importance of each selected variable, and its contribution with respect to a sample group. Some plots display specified components (`plotVar, plotLoadings`) while others can also aggregate the similarities between variables across all components (`cim, networks`).

`plotVar` (Correlation circle plots). Correlation circle plots display the correlation between variables (biological features) and latent components. Each variable coordinate is defined as the Pearson correlation between the original data and a latent component (see González et al. 2012 for a detailed description). Correlation circle plots are particularly useful to visualise the contribution of each variable to define each component (variable close to the large circle of radius 1), as well as the correlation structure between variables (clusters of variables). The cosine angle between any two points represent the correlation (negative, positive or null) between two variables.

`network` (Relevance networks) Relevance networks represent the correlation structure between variables of different types. The function avoids the intensive computation of Pearson correlation matrices on large data set by calculating instead a pair-wise similarity matrix directly obtained from the latent components of the integrative approaches (CCA, PLS, block.pls methods). The similarity value between a pair of variables is obtained by calculating the sum of the correlations between the original variables and each of the latent components of the model. The values in the similarity matrix can be seen as a robust approximation of the Pearson correlation (see González et al. 2012 for a mathematical demonstration and exact formula). The advantage of relevance networks is their ability to simultaneously represent positive and negative correlations, which are missed by methods based on Euclidian distances or mutual information. Those networks are bipartite and thus only a link between two variables of different types can be represented. The network can be saved in a .glm format using the `igraph` package, the function `write.graph` and extracting the output `$gR`, see details in our Sweave workflow for `DIABLO`.

`cim` (Clustered Image Maps). The plot visualises the distances between two types of variables (two-‘omics integration), or the distances between variables and samples (single ‘omics supervised and P- integration). CIM is based on a hierarchical clustering simultaneously operating on the rows and columns of the selected variables in the original data for the latter, and on the similarity matrix defined in the network visualisation for the former. By default we use Euclidian distance and complete linkage method but other distances and methods are proposed. For N-integration, the function `cimDiablo` represents the selected features from the different data sets.

`plotLoadings` (Loading plots). The plot represents the loading weights of each variable (selected) on each dimension of the multivariate model. Most important variables (according to the absolute value of their coefficients) are ordered from bottom to top. For supervised analyses, colours indicate the class for which the mean (or median) expression value is the highest or the lowest for each feature (`contrib = ‘max’` or `‘min’`). This graphical output enables to better characterise the molecular signature, especially when interpreted in conjunction with the sample plot (see Fig. 1).

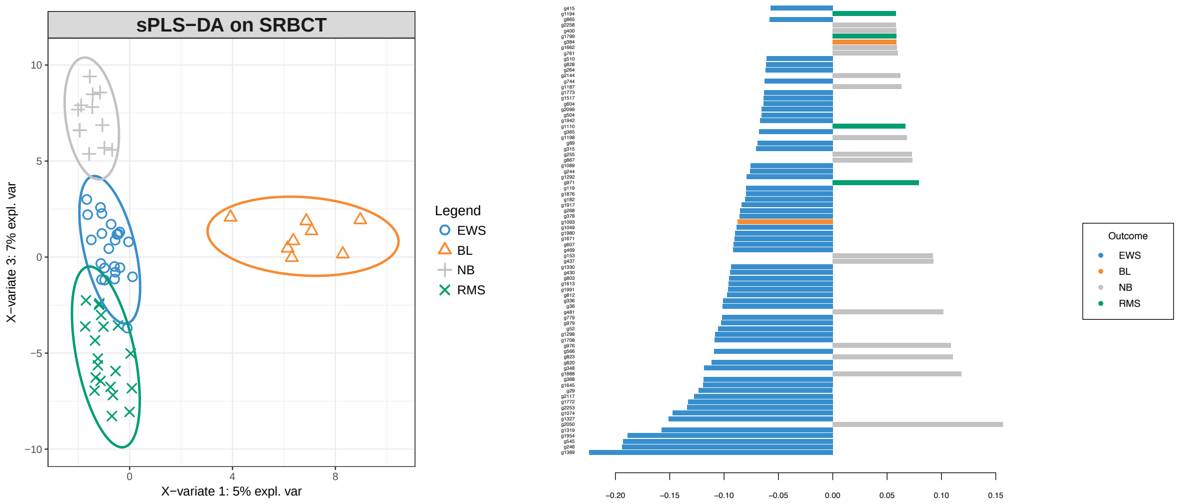

Figure 1: **Example of sample plot with `plotIndiv` and loading plot with `plotLoadings` with sPLS-DA**. The loading plot (right) represents the top 80 genes selected on the second component of the sPLS-DA model. Colors indicate the tumour subtype where the mean expression levels of the gene is maximal. More details in the 'Results' Section R and sweave code 3.4.

2.3. Additional graphical outputs

Both `plotIndiv` and `plotVar` offer usual plot arguments to display symbols, colours and legend, with graphic styles include `ggplot2` (by default), `graphics`, `lattice` and 3D plots.

Other graphical outputs are available in `mixOmics` to visualise the classification performance of the multivariate models using the generic function `plot`. The listing of the functions for each framework presented in our main article are summarised in Fig 2.

Finally, additional graphical outputs or variants for the frameworks `DIABLO` and `MINT` are described below (`plotDiablo, circosPlot`).

3. Single 'omics supervised multivariate analysis with PLS-DA and sPLS-DA

Linear Discriminant Analysis (LDA) and Projection to Latent Structure (PLS, Wold 1966) are popular multivariate methods for supervised analyses. In `mixOmics` we mainly focus on PLS methods for their flexibility to solve a variety of analytical problems (Boulesteix and Strimmer, 2007). PLS regression (Wold, 1966) was originally developed for unsupervised analysis to integrate two data sets with continuous variables, measured on the same observations or samples. We briefly present the supervised version of PLS, called PLS-Discriminant Analysis (PLS-DA, Nguyen and Rocke 2002; Barker and Rayens 2003). PLS-DA is natural extension of PLS that substitutes one of the data set for a dummy indicator matrix Y. PLS-DA fits a classifier multivariate model that assigns samples into known classes, with the ultimate aim to predict the classes of external test samples where the outcome might be unknown.

3.1. PLS - Discriminant Analysis

PLS-DA is an iterative method that constructs H successive artificial (latent) components $t_h = X_h a_h$ and $u_h = Y_h b_h$ for $h = 1, .., H$, where the hth component t_h (respectively u_h) is a linear combination of the X (Y) variables. H denotes the dimension of the PLS-DA model. The weight coefficient vector a_h (b_h) is the loading vector that indicates the *importance* of each variable to define the component. For each dimension

	functions	PLS-DA	sPLS-DA	DIABLO	sparse DIABLO	MINT	sparse MINT
function call		`plsda`	`splsda`	`block.plsda`	`block.splsda`	`mint.plsda`	`mint.splsda`
parameters		ncomp	ncomp keepX	design ncomp	design ncomp keepX	ncomp	ncomp keepX
performance	`tune, plot.tune`		✓		✓		✓
	`perf, plot.perf`	✓	✓	✓	✓	✓	✓
	`auroc`	✓	✓	✓	✓	✓	✓
sample plot	`plotIndiv`	✓	✓	✓	✓	✓	✓
	`plotArrow`	✓	✓	✓	✓	✓	✓
	`plotDiablo`			✓	✓		
variable plot	`plotVar`	✓	✓	✓	✓	✓	✓
	`plotLoadings`	✓	✓	✓	✓	✓	✓
	`circosPlot`			✓	✓		
	`cim`	✓	✓	✓	✓	✓	✓
	`network`	✓	✓	✓	✓	✓	✓
variable list	`selectVar`	✓	✓	✓	✓	✓	✓

Figure 2: **Summary of the main `mixOmics` S3 functions for supervised multivariate analyses.**

$h = 1, \ldots, H$, PLS-DA seeks to maximize

$$\max_{(a_h, b_h)} \text{cov}(X_h a_h, Y_h b_h), \qquad \text{s.t.} \quad ||a_h||_2 = ||b_h||_2 = 1 \tag{2}$$

where X_h, Y_h are the residual (deflated) matrices extracted from each iterative linear regression (see Lê Cao et al. 2011 for more details). The PLS-DA model assigns to each sample i a pair of H scores (t_h^i, u_h^i) which effectively represents the projection of that sample into the X- or Y- space spanned by those PLS components. As $H << P$, the projection space is small, allowing for dimension reduction as well as insightful sample plot representation. Note that the projection into the Y-space is not used for Discriminant Analysis with PLS-DA.

3.2. Variable selection with sparse PLS-DA

We developed a *sparse* version of PLS-DA (Lê Cao et al., 2011) which includes an ℓ^1 penalisation (Tibshirani, 1996) on the loading vector a_h to shrink some coefficients to zero. Thus, for each dimension $h = 1, .., H$, sPLS-DA solves:

$$\max_{(a_h, b_h)} \text{cov}(X_h a_h, Y_h b_h), \qquad \text{s.t.} \quad ||a_h||_2 = ||b_h||_2 = 1 \text{ and } ||a_h||_1 \leq \lambda_h \tag{3}$$

where λ_h is a non negative parameter that controls the amount of shrinkage in a_h. The component scores $t_h = X_h a_h$ are now defined on a small subset of variables with non-zero coefficients, leading to variable

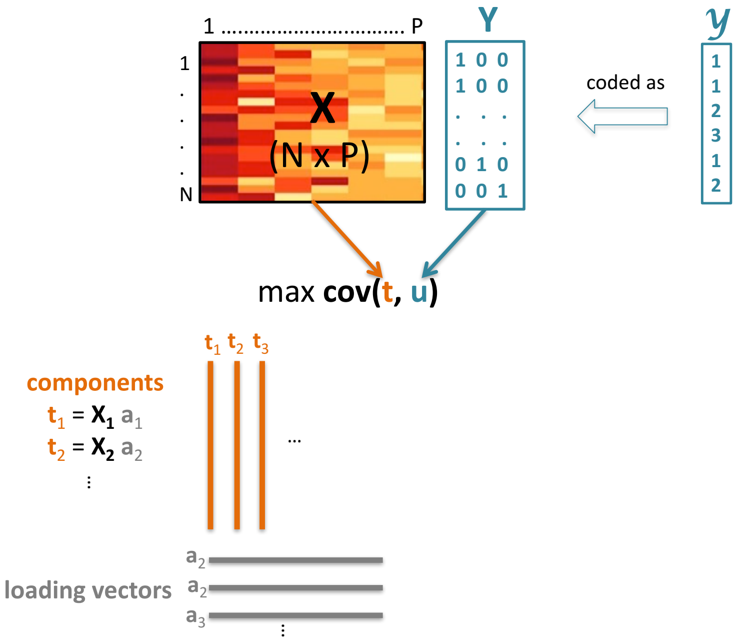

Figure 3: **Example of data matrix decomposition for single 'omics analysis with PLS-DA.** The predictor matrix X is decomposed into a set of components $(t_1, \ldots, t_H)$ and associated loading vectors $(a_1, \ldots, a_H)$, $\boldsymbol{Y}$ is the outcome coded as a dummy indicator matrix as described in Table 1 and combined linearly (see exact formula in Equation (2)). $\boldsymbol{X_h}$ is the deflated (residual) matrix starting with $\boldsymbol{X_1} = \boldsymbol{X}$, for $h = 1 \ldots H$. H is the dimension of the model - or the number of components.

selection that aims to optimally maximise the discrimination between the K outcome classes in Y. In `mixOmics` we use soft-thresholding to improve usability by replacing the λ_h parameter by the number `keepX` of features to select on each dimension (see 'Choice of parameters' Section in the main article).

3.3. Extensions of PLS-DA for repeated measurements and 16S microbiome data

PLS-DA and sPLS-DA were extended to account for repeated measurement designs, as described in Liquet et al. (2012) by specifying the argument `multilevel` in the `plsda` and `splsda` functions.

Recent extensions in the package also include sPLS-DA analysis to identify microbial communities for 16S data with an additional `logratio` argument to account for compositional data in microbiome experiment (Lê Cao et al. 2016, see also our `mixMC` framework in `www.mixOmics.org/mixMC`).

3.4. Workflow for PLS-DA analysis

We provide a full R and Sweave workflow electronically on our website tutorial page or at this link for all frameworks.

4. N-integration across multiple 'omics data sets with DIABLO

The integration of multiple 'omics datasets measured on the same N biological samples (Figure 1 in main article) is based on a variant of the multivariate methodology Generalised Canonical Correlation Analysis (GCCA, Tenenhaus and Tenenhaus 2011; Tenenhaus et al. 2014), which, contrary to what its name suggests, generalises PLS for N-integration. Multiple data integration is a challenging task, as the analysis can be strongly affected by artefacts of the 'omics technological platforms or by variation between manufacturers,

despite being measured on the same biological samples. Our recent development DIABLO further improved the implementation of GCCA to include variable selection in a supervised framework and in a user-friendly manner (Günther et al., 2014; Singh et al., 2016). We have applied DIABLO to integrate different 'omics experiments arising from different 'omics platforms (*e.g.* transcriptomics, proteomics in Günther et al. 2014, as well as mRNA, proteomics, miRNA and methylation data in Singh et al. 2016), to identify correlated or *co-expressed* 'omics variables that also explain the outcome of interest.

4.1. DIABLO method

We denote Q 'omics data sets $X^{(1)}(N \times P_1)$, $X^{(2)}(N \times P_2)$, ..., $X^{(Q)}(N \times P_Q)$ measuring the expression levels of P_q 'omics variables on the same N biological samples, $q = 1, \dots, Q$. GCCA solves for each component $h = 1, \dots, H$:

$$\max_{a_h^{(1)},\dots,a_h^{(Q)}} \sum_{q,j=1,q\neq j}^{Q} c_{q,j} \; \mathrm{cov}(X_h^{(q)} a_h^{(q)}, X_h^{(j)} a_h^{(j)}), \qquad \text{s.t.} \quad ||a_h^{(q)}||_2 = 1 \text{ and } ||a_h^{(q)}||_1 \leq \lambda^{(q)} \tag{4}$$

where $\lambda^{(q)}$ is the penalisation parameter, $a_h^{(q)}$ is the loading vector on component h associated to the residual (deflated) matrix $X_h^{(q)}$ of the data set $X^{(q)}$, and $C = \{c_{q,j}\}_{q,j}$ is the design matrix. C is a $Q \times Q$ matrix that specifies whether datasets should be correlated and includes values between zero (datasets are not connected) and one (datasets are fully connected). Thus, it is possible to constraint the model to only take into account specific pairwise covariances by setting the design matrix (see Tenenhaus et al. 2014 for more details). Such design thus enables to model a particular association between pairs of 'omics data, as expected from prior biological knowledge or experimental design. DIABLO Discriminant Analysis in mixOmics extends (4) to a supervised framework by replacing one data matrix $X^{(q)}$ with the outcome dummy matrix Y.

Choice of the design matrix There are different strategies to specify the design matrix. One can define the design matrix based on prior biological knowledge (*e.g.* proteomics and transcriptomics 'should be' highly correlated), or based on a data-driven approach. A preliminary multivariate method integrating two data sets at a time (e.g. pls) can assess the common information between data sets in an unsupervised analysis, while the performance evaluation of different designs can guide the choice when seeking for a predictive multi-'omics model. Our experience has shown that a trade-off between maximising correlation between datasets and maximising the discrimination of the outcome was required. Assume that $X^{(Q)}$ is set to the outcome dummy indicator matrix. A *full design*, where $c_{q,j} = 1$ for all $q \neq j$, maximises the correlation between datasets while a *null design*, where $c_{q,j} = 0$ for $q, j < Q$ and $c_{q,j} = 1$ for $q, j = Q$, maximises the separation between the outcome classes. In practice, we found that a *full weighted design* where $c_{q,j} = 0.1$ between data matrices and 1 for the outcome led to such trade-off (a correlated and discriminant molecular signature), see Figure 4.

4.2. Specific outputs to visualise multiple 'omics data sets integration

Several types of graphical outputs are available to support interpretation of the statistical results. To represent samples, plotIndiv displays component scores from each 'omics data set individually. Such type of plot enable to visualise the agreement between all data sets at the sample level. The plotArrow function also enables similar visualisation (see Section 2). The function plotDiablo is a matrix scatterplot of the components from each data set for a given dimension; it enables to check whether the pairwise correlation between two 'omics has been modelled according to the design.

The function circosPlot shows pairwise correlations among the selected variables across all data sets. Variables are represented on the side of the circos plot, where colours indicate the type of data, and external (optional) lines display the expression levels with respect to each outcome category. circosPlot implement an extension of the method used in plotVar, cim and network described in González et al. (2012).

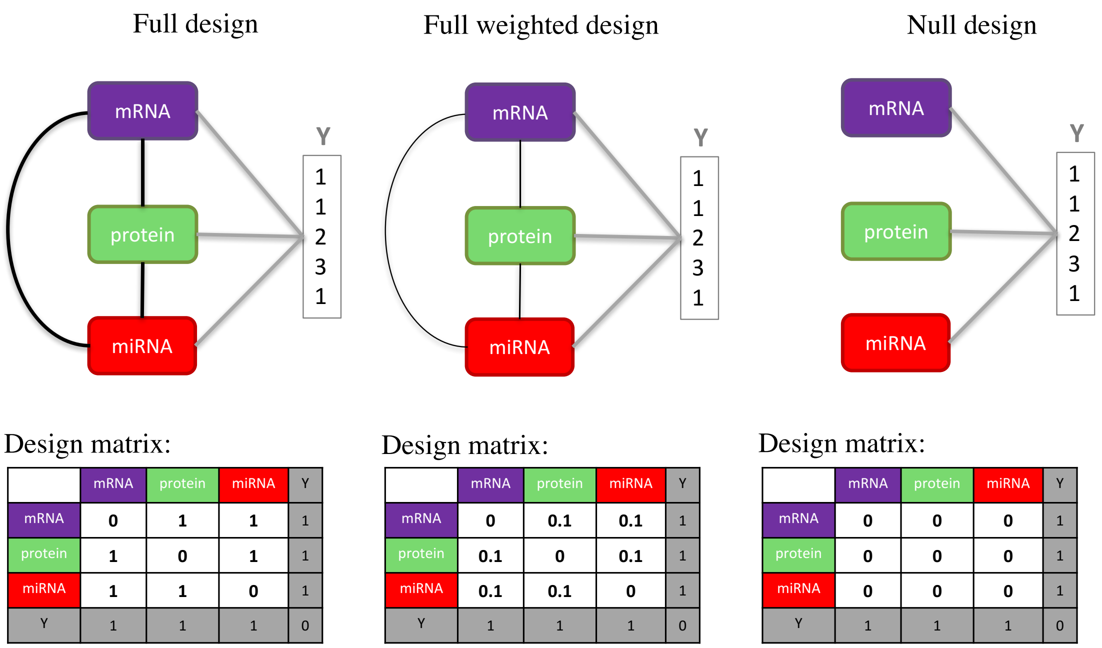

Figure 4: **Example of different design matrices in `DIABLO` for the multi-'omics breast cancer study** (illustrated in the main article). Links or cells in grey are added by default in the `DIABLO` function `block.plsda` and `block.splsda` and do not need to be specified by the user.

4.3. Workflow for `DIABLO` analysis

We provide a full R and Sweave workflow electronically on our website tutorial page or at this link for all frameworks.

5. *P*-integration across independent data sets with MINT

The integration of independent data sets measured on the same common P features under similar conditions or treatments (Figure 1 in main article) is a useful approach to increase sample size and gain statistical power. In this context, the challenge is to accommodate for systematic differences that arise due to differences between protocols, geographical sites or the use of different technological platforms to generate the same type of 'omics data (e.g. transcriptomics). The systematic unwanted variation, also called 'batch-effect', often acts as a strong confounder in the statistical analysis and may lead to spurious results and conclusions if it is not accounted for in the statistical model. Here we briefly introduce `MINT`, a PLS-based supervised method that integrates independent studies in a classification framework.

5.1. `MINT` method

`MINT` (Rohart et al., 2017) is an extension of the multi-group PLS framework (mgPLS, Eslami et al. 2013, 2014), where 'groups' represent independent studies, to a supervised framework with variable selection.

MINT seeks for a common projection space across all studies, defined on a small subset of discriminative variables that consistently discriminate the outcome classes across studies.

In `MINT`, we combine M datasets denoted $X^{(1)}(N_1 \times P)$, $X^{(2)}(N_2 \times P)$, ..., $X^{(M)}(N_M \times P)$ measured on the same P predictors but from independent studies, with $N = \sum_{m=1}^{M} N_m$. Each data set $X^{(m)}$, $m = 1, \ldots, M$, has an associated dummy indicator outcome $Y^{(m)}$ in which all K classes are represented. We denote X $(N \times P)$ and Y $(N \times K)$ the concatenation of all $X^{(m)}$ and $Y^{(m)}$ respectively. In our `MINT` framework, each variable from the datasets $X^{(m)}$ and $Y^{(m)}$ is centered and scaled within each study m. For each component h, `MINT` solves :

$$\max_{a_h, b_h} \sum_{m=1}^{M} N_m \text{ cov}(X_h^{(m)} a_h, Y_h^{(m)} b_h), \qquad \text{s.t.} \quad ||a_h||_2 = 1 \text{ and } ||a_h||_1 \leq \lambda \tag{5}$$

where a_h and b_h are the global loadings vectors common to all studies, $t_h^{(m)} = X_h^{(m)} a_h$ and $u_h^{(m)} = Y_h^{(m)} b_h$ are the partial PLS-components that are study specific. Residual (deflated) matrices are calculated for each iteration of the algorithm based on the global components and loading vectors (see Rohart et al. 2017). Thus the `MINT` algorithm models the study structure during the integration process. The penalisation parameter λ controls the amount of shrinkage and thus the number of non zero weights in the global loading vector a. Similarly to sPLS-DA (Section 3.2) `MINT` selects a combination of features on each PLS-component.

5.2. Leave-One-Group-Out Cross-Validation for performance assessment

In `MINT` we take advantage of the independence between studies to evaluate the performance based on a novel CV technique called 'Leave-One-Group-Out Cross-Validation' (Rohart et al., 2017). LOGOCV performs CV where each study m is left out once. The aim is to reflect a realistic prediction of independent external studies. Note that LOGOCV cannot be repeated (no `nrepeat` argument) as the partitioning is not random.

5.3. Specific graphical outputs for `MINT`

The set of partial components $t_h^{(m)}, h = 1, ..., H$ provides outputs specific to each study m in `plotIndiv`. The samples plots enable to perform a quality control step to identify studies that cluster outcome classes differently to other studies (i.e. 'outlier' studies). The function `plotLoadings` displays the coefficients weights of the features globally selected by the model but represented individually in each study to visualise potential discrepancies between studies. Visualisation of the global loading vectors is also possible (argument `study = 'all.partial'` or `'global'`). Note the projection into the Y-space is not useful in `MINT`.

5.4. Workflow for `MINT` analysis

We provide a full R and Sweave workflow electronically on our website tutorial page or at this link for all frameworks.

6. Computational time for large data sets

We report some examples of computational time for very large data sets we recently analysed with `mixOmics`. Note that for most analyses, we would recommend filtering the data sets, as detailed in Section 'Data input' in the main article for more tractable tuning of the `ncomp` and `keepX` parameters.

Table 4: Example of runtime for very large data sets analysed in `mixOmics`. Tuning and performance assessments were performed with 5-fold CV for single 'omics and N-integration, or LOGOCV for P-integration (Rohart et al. 2017, Singh et al. 2016, cluster with 10 cpus and 50 Gb RAM).

Framework	Single 'omics sPLS-DA		N-integration DIABLO		P-integration MINT	
Data	HNSCC		Asthma (2 omics)		Stem Cell (8 studies)	
N	60		194		210	
P	$82,132$		$30,000; 30,000$		$13,313$	
function	`tune`	`perf`	`tune`	`perf`	`tune`	`perf`
#fold CV (repeated)	5(10)	5(10)	5(1)	5(10)	LOGOCV	LOGOCV
ncomp	5	3	2	2	2	2
grid length per component	40	-	22^2	-	100	-
#cpu	10	10	10	10	1	1
runtime	15min	6min	19min	3min	17min	12sec

References

Barker, M. and Rayens, W. (2003). Partial least squares for discrimination. *Journal of chemometrics*, 17(3):166–173.

Boulesteix, A.-L. and Strimmer, K. (2007). Partial least squares: a versatile tool for the analysis of high-dimensional genomic data. *Brief. Bioinform.*, 8(1):32–44.

Eslami, A., Qannari, E. M., Kohler, A., and Bougeard, S. (2013). Multi-group PLS Regression: Application to Epidemiology. In *New Perspectives in Partial Least Squares and Related Methods*, pages 243–255. Springer.

Eslami, A., Qannari, E. M., Kohler, A., and Bougeard, S. (2014). Algorithms for multi-group PLS. *J. Chemometrics*, 28(3):192–201.

González, I., Lê Cao, K.-A., Davis, M. J., Déjean, S., et al. (2012). Visualising associations between paired 'omics' data sets. *BioData mining*, 5(1):19.

Günther, O. P., Shin, H., Ng, R. T., McMaster, W. R., McManus, B. M., Keown, P. A., Tebbutt, S. J., and Lê Cao, K.-A. (2014). Novel multivariate methods for integration of genomics and proteomics data: applications in a kidney transplant rejection study. *Omics: a journal of integrative biology*, 18(11):682–695.

Lê Cao, K.-A., Boitard, S., and Besse, P. (2011). Sparse PLS Discriminant Analysis: biologically relevant feature selection and graphical displays for multiclass problems. *BMC bioinformatics*, 12(1):253.

Lê Cao, K.-A., Lakis, V. A., Bartolo, F., Costello, M.-E., Chua, X.-Y., Brazeilles, R., and Rondeau, P. (2016). Mixmc: Multivariate insights into microbial communities. *PloS one*, 11(8):e0160169.

Lê Cao, K.-A., Rohart, F., Gonzalez, I., Déjean, S., Gautier, B., Bartolo, F., Monget, P., Coquery, J., Yao, F., and Liquet., B. (2017). *mixOmics: Omics Data Integration Project*. R package version 6.2.0.

Liquet, B., Lê Cao, K.-A., Hocini, H., and Thiébaut, R. (2012). A novel approach for biomarker selection and the integration of repeated measures experiments from two assays. *BMC bioinformatics*, 13:325.

Nguyen, D. V. and Rocke, D. M. (2002). Multi-class cancer classification via partial least squares with gene expression profiles. *Bioinformatics*, 18(9):1216–1226.

Rohart, F., Eslami, A., Matigian, N., Bougeard, S., and Lê Cao, K.-A. (2017). Mint: A multivariate integrative approach to identify a reproducible biomarker signature across multiple experiments and platforms. *BMC Bioinformatics*, 18(128).

Singh, A., Gautier, B., Shannon, C. P., Vacher, M., Rohart, F., Tebutt, S. J., and Lê Cao, K.-A. (2016). Diablo-an integrative, multi-omics, multivariate method for multi-group classification. *bioRxiv*, 067611.

Tenenhaus, A., Philippe, C., Guillemot, V., Lê Cao, K.-A., Grill, J., and Frouin, V. (2014). Variable selection for generalized canonical correlation analysis. *Biostatistics*, 15(3):569 – 83.

Tenenhaus, A. and Tenenhaus, M. (2011). Regularized generalized canonical correlation analysis. *Psychometrika*, 76(2):257–284.

Tibshirani, R. (1996). Regression shrinkage and selection via the lasso. *Journal of the Royal Statistical Society. Series B (Methodological)*, pages 267–288.

Wold, H. (1966). Estimation of principal components and related models by iterative least squares. *J. Multivar. Anal.*, pages 391–420.

Wold, H. (1975). *Path models with latent variables: The NIPALS approach.* Acad. Press.
