# Supplementary material for: mixOmics: An R package for ‘omics feature selection and multiple data integration
Source: PLoS Comput Biol. 2017 Nov 3;13(11):e1005752. doi: 10.1371/journal.pcbi.1005752 (PMC5687754; doi:10.1371/journal.pcbi.1005752)
Supplement: S1 Appendix — (ZIP) [file pcbi.1005752.s002.zip › mixOmicsRscripts/DIABLO-analysis.pdf]

E2. N-integration across multiple 'omics data sets with DIABLO

Contents

E2 N-integration across multiple 'omics data sets with DIABLO . . . 1
E2.1 Data . . . 1
E2.2 Parameter choice . . . 2
E2.3 Final model . . . 4
E2.4 Sample plots . . . 5
E2.5 Variable plots . . . 8
E2.6 Performance of the model . . . 13
E2.7 Prediction on an external test set. . . . 14
E2.8 Session information of this Sweave code . . . 15

We first load mixOmics:

```
> library(mixOmics)
```

E2.1. Data

Human breast cancer is a heterogeneous disease in terms of molecular alterations, cellular composition, and clinical outcome. Breast tumours can be classified into several subtypes, according to levels of mRNA expression (Sørlie et al., 2001). Here we consider a subset of data generated by The Cancer Genome Atlas Network (Cancer Genome Atlas Network et al., 2012). For the package, data were normalised and drastically prefiltered for illustrative purposes. The data were divided into a training set with a subset of 150 samples from the mRNA, miRNA and proteomics data, and a test set including 70 samples, but only with mRNA and miRNA data (proteomics missing). The aim of the N-integration analysis is to identify a highly correlated multi-'omics signature discriminating the breast cancer subtypes Basal, Her2 and LumA (Singh et al., 2016).

The data for DIABLO are set as a list of data matrices matching the same samples in rows.

```
> data('breast.TCGA')
> # extract training data
> data = list(mRNA = breast.TCGA$data.train$mrna,
+             miRNA = breast.TCGA$data.train$mirna,
+             proteomics = breast.TCGA$data.train$protein)
> # check dimension
> lapply(data, dim)
```

```
$mRNA
[1] 150 200

$miRNA
[1] 150 184

$proteomics
[1] 150 142
```

```
> # outcome
> Y = breast.TCGA$data.train$subtype
> summary(Y)
```

```
Basal  Her2  LumA
   45    30    75
```

E2.2. Parameter choice

Here we choose a design where all blocks (data sets) are connected with a link of 0.1 (see Supplemental Information).

```
> design = matrix(0.1, ncol = length(data), nrow = length(data),
+                 dimnames = list(names(data), names(data)))
> diag(design) = 0
> design
```

```
           mRNA miRNA proteomics
mRNA        0.0   0.1        0.1
miRNA       0.1   0.0        0.1
proteomics  0.1   0.1        0.0
```

First, we fit a DIABLO model without variable selection to assess the global performance and choose the number of components for the final DIABLO model. The function `perf` is run with 10-fold cross validation repeated 10 times.

The elapsed reuning time is reported in seconds.

```
> sgccda.res = block.splsda(X = data, Y = Y, ncomp = 5,
+                           design = design)
> set.seed(123) # for reproducibility, only when the `cpus' argument is not used
> t1 = proc.time()
> perf.diablo = perf(sgccda.res, validation = 'Mfold', folds = 10, nrepeat = 10)
> t2 = proc.time()
> running_time = t2 - t1; running_time
```

```
   user  system elapsed
 58.929   0.924  62.011
```

```
> #perf.diablo  # lists the different outputs
> plot(perf.diablo)
>
```

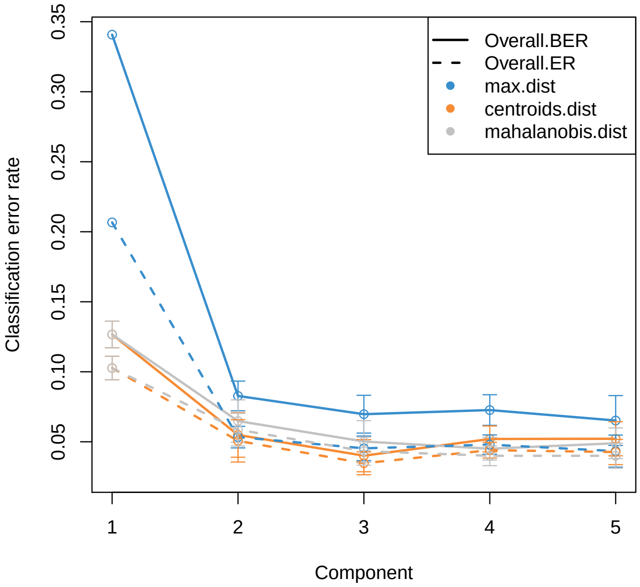

From the performance plot above we observe that both overall and balanced error rate (BER) decrease from 1 to 2 components. The standard deviation indicates a potential slight gain in adding more components. The centroids.dist distance seem to give the best accuracy (see Supplemental Material (Rohart et al., 2017)). Considering this distance and the BER, the output `$choice.ncomp` indicates an optimal number of components = 2 for the final DIABLO model.

```
> perf.diablo$choice.ncomp$WeightedVote
```

```
            max.dist centroids.dist mahalanobis.dist
Overall.ER         2              3                3
Overall.BER        5              2                4
```

```
> ncomp = perf.diablo$choice.ncomp$WeightedVote["Overall.BER", "centroids.dist"]
```

Now that the number of components is chosen, the next step is to choose the optimal number of variables to select in each data set using the `tune.block.splsda` function. We provide a grid of `keepX` values for each type of 'omics. Note that we set the grid to favour a small (but not too small) signature while allowing to obtain a sufficient number of variables for downstream validation / interpretation.

Here we have saved the results into a RData object, so this code will not be run during the Sweave compilation. The elapsed running time is indicated in seconds. The function `tune` is run with 10-fold cross validation, but repeated only once. Note that for a more thorough tuning process, provided sufficient computational time, we could increase the `nrepeat` argument.

```
> #set.seed(123) # for reproducibility, only when the `cpus' argument is not used
> test.keepX = list (mRNA = c(5:9, seq(10, 18, 2), seq(20,30,5)),
+                    miRNA = c(5:9, seq(10, 18, 2), seq(20,30,5)),
+                    proteomics = c(5:9, seq(10, 18, 2), seq(20,30,5)))
> t1 = proc.time()
```

```
> tune.TCGA = tune.block.splsda(X = data, Y = Y, ncomp = ncomp,
+                               test.keepX = test.keepX, design = design,
+                               validation = 'Mfold', folds = 10, nrepeat = 1,
+                               dist = "centroids.dist", cpus = 2)
> t2 = proc.time()
> running_time = t2 - t1; running_time
> list.keepX = tune.TCGA$choice.keepX
> list.keepX
```

```
    user   system  elapsed
   3.366    0.144 1052.726
```

The number of features to select on each component is returned in `tune.TCGA$choice.keepX`:

```
> list.keepX = tune.TCGA$choice.keepX
> tune.TCGA$choice.keepX
```

```
$mRNA
[1] 16  7

$miRNA
[1] 18  5

$proteomics
[1] 5 5
```

E2.3. Final model

The final `DIABLO` model is run as:

```
> sgccda.res = block.splsda(X = data, Y = Y, ncomp = ncomp,
+                           keepX = list.keepX, design = design)
> #sgccda.res   # lists the different functions of interest related to that object
```

The warning message informs that the outcome Y has been included automatically in the design, so that the covariance between each block's component and the outcome is maximised, as shown in the final design output:

```
> sgccda.res$design
```

```
           mRNA miRNA proteomics Y
mRNA        0.0   0.1        0.1 1
miRNA       0.1   0.0        0.1 1
proteomics  0.1   0.1        0.0 1
Y           1.0   1.0        1.0 0
```

The selected variables can be extracted with the function `selectVar`, for example in the mRNA block, along with their loading weights:

```
> # mRNA variables selected on component 1
> selectVar(sgccda.res, block = 'mRNA', comp = 1)
```

```
$mRNA
$mRNA$name
 [1] "ZNF552"  "KDM4B"   "CCNA2"   "LRIG1"   "PREX1"   "FUT8"    "C4orf34"
 [8] "TTC39A"  "ASPM"    "SLC43A3" "MEX3A"   "SEMA3C"  "E2F1"    "STC2"
[15] "FMNL2"   "LMO4"

$mRNA$value
           value.var
ZNF552  -0.483342279
KDM4B   -0.408455112
CCNA2    0.316039919
LRIG1   -0.301880859
PREX1   -0.300287075
FUT8    -0.282105086
C4orf34 -0.269503106
TTC39A  -0.258567084
ASPM     0.173086718
SLC43A3  0.163846613
MEX3A    0.144064728
SEMA3C  -0.128643646
E2F1     0.059131148
STC2    -0.036197206
FMNL2    0.018170346
LMO4     0.006438995


$comp
[1] 1
```

Also note that the stability of the selected variables can be extracted from the `perf` function, similar to the example given in the PLS-DA analysis (Electronic file in (Rohart et al., 2017)).

E2.4. Sample plots

`plotDIABLO` is a diagnostic plot to check whether the correlation between components from each data set was maximised as specified in the design matrix. We specify the dimension to be assessed with the `ncomp` argument.

```
> plotDiablo(sgccda.res, ncomp = 1)
```

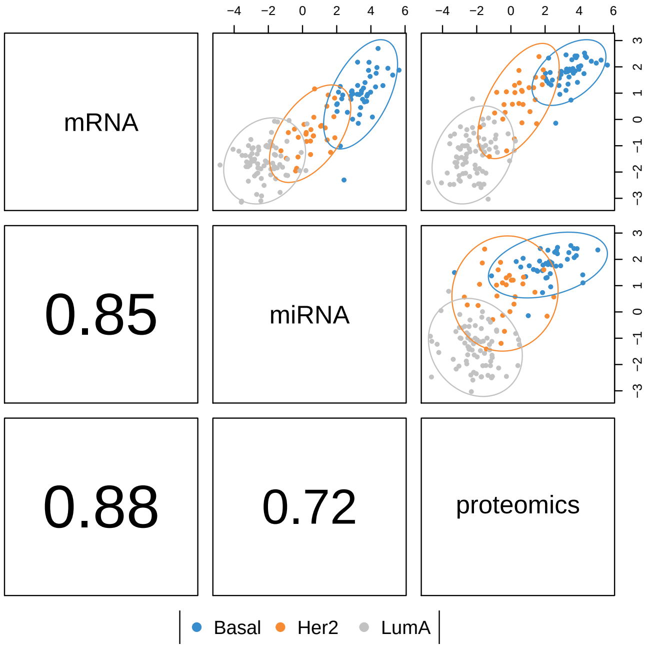

The set of first components from all data set are highly correlated. The colors and ellipses represent the sample subtypes and indicate the discriminative power of each component to separate the different tumour subtypes.

The sample plot with the `plotIndiv` function projects each sample into the space spanned by the components from each block. The optional argument `blocks` can output a specific data set. Ellipse plots are also available (argument `ellipse = TRUE`). This type of graphic allows us to better understand the information extracted from each data set and its discriminative ability.

```
> plotIndiv(sgccda.res, ind.names = FALSE, legend = TRUE,  style="ggplot2")
```

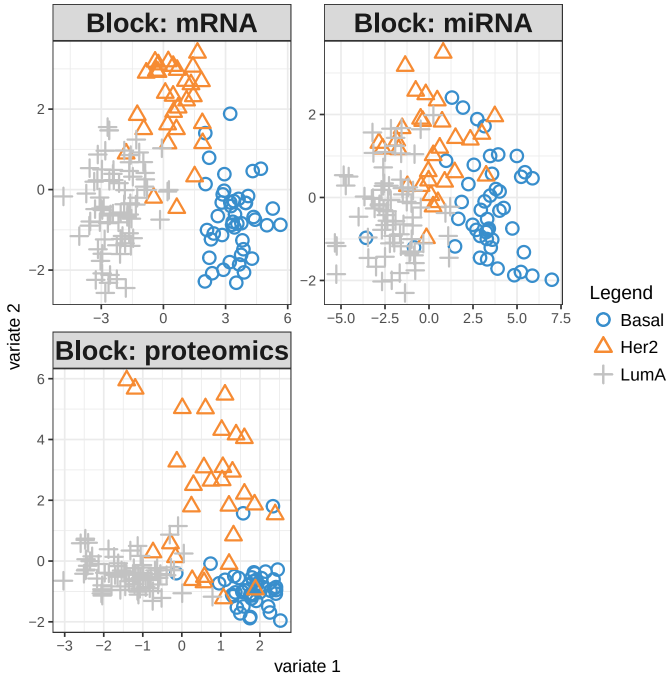

In the arrow plot below, the start of the arrow indicates the centroid between all data sets for a given sample and the tip of the arrow the location of the same sample in each block. Such graphic highlights the agreement between all data sets at the sample level when modelled with `DIABLO`.

```
> plotArrow(sgccda.res, ind.names = FALSE, legend = TRUE, title = 'DIABLO')
```

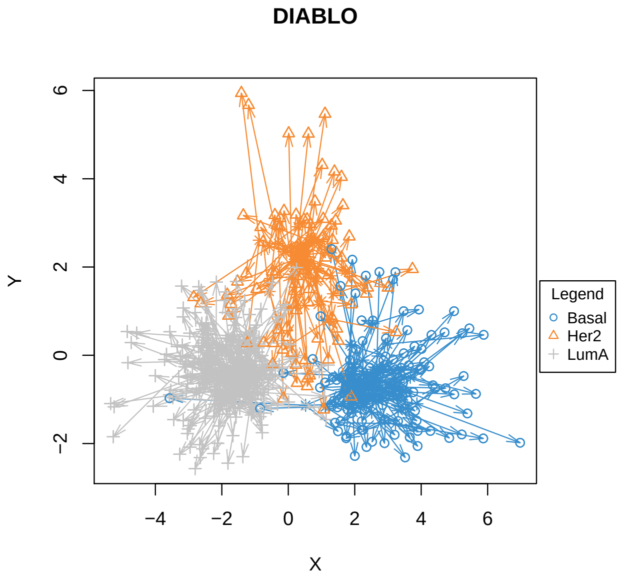

E2.5. Variable plots

Several graphical outputs are available to visualise and mine the associations between the selected variables.

The correlation circle plot highlights the contribution of each selected variable to each component. Important variables should be close to the large circle, see González et al. 2012 for more details. `plotVar` displays the variables from all blocks, selected on component 1 and 2. Clusters of points indicate a strong correlation between variables. For better visibility we choose to hide the variable names.

```
> plotVar(sgccda.res, var.names = FALSE, style = 'graphics', legend = TRUE,
+         pch = c(16, 17, 15), cex = c(2,2,2), col = c('darkorchid', 'brown1', 'lightgreen'))
```

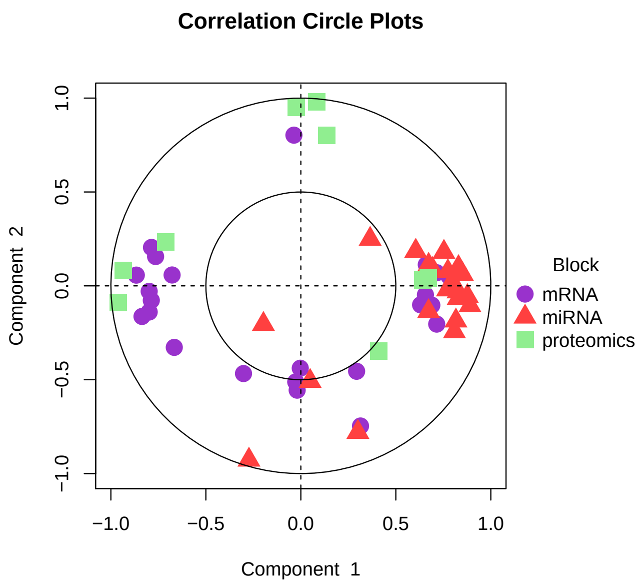

We can also only display the variables selected on a specific component, here component 1:

```
> plotVar(sgccda.res, var.names = FALSE, style = 'graphics', legend = TRUE,
+         pch = c(16, 17, 15), cex = c(2,2,2), col = c('darkorchid', 'brown1', 'lightgreen'),
+         comp.select = 1)
```

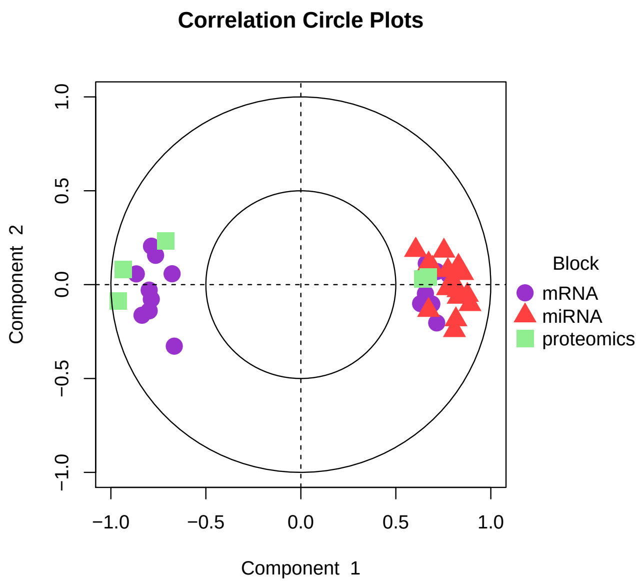

The circos plot represents the correlations between variables of different types, represented on the side quadrants. Several display options are possible, to show within and between connexions between blocks, expression levels of each variable according to each class (argument `line = TRUE`). The circos plot is built based on a similarity matrix, which was extended to the case of multiple data sets from González et al. 2012.

```
> circosPlot(sgccda.res, cutoff = 0.7, line = TRUE,
+            color.blocks= c('darkorchid', 'brown1', 'lightgreen'),
+            color.cor = c("chocolate3","grey20"), size.labels = 1.5)
```

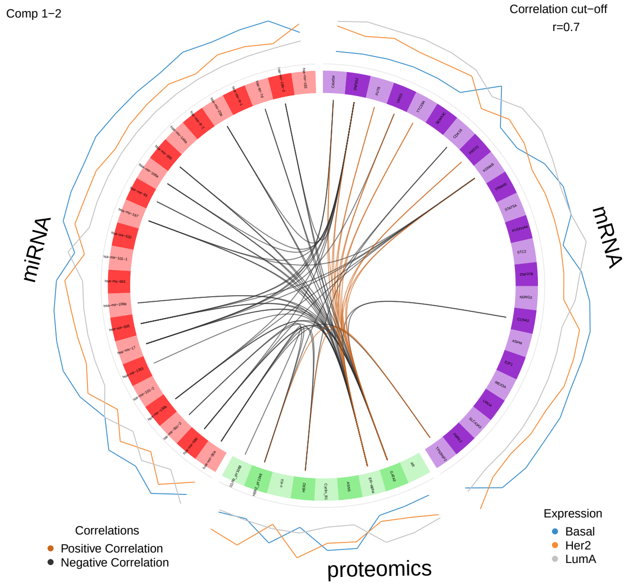

Another visualisation of the correlation between the different types of variables is the relevance network, which is also built on the similarity matrix. Each color represents a type of variable. A threshold can also be set using the argument `cutoff`.

Note that sometimes the output may not show with Rstudio because of margin issues. The plot can be saved as an image using the argument `save` and `name.save`. An `interactive` argument is also available for the `cutoff` argument, see details in `?network`.

```
> network(sgccda.res, blocks = c(1,2,3),
+         color.node = c('darkorchid', 'brown1', 'lightgreen'), cutoff = 0.4)
```

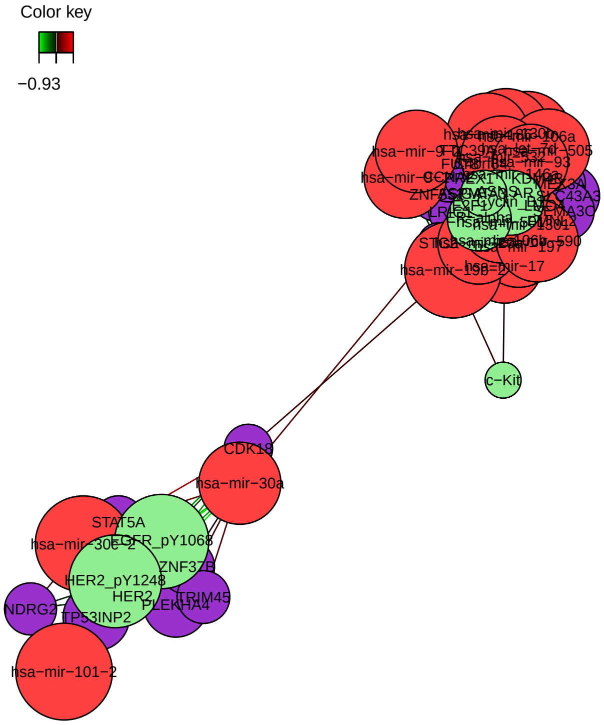

The network can be saved in a .gml format to be input into the software Cytoscape, using the R package igraph

```
> # not run
> library(igraph)
> my.network = network(sgccda.res, blocks = c(1,2,3),
+         color.node = c('darkorchid', 'brown1', 'lightgreen'), cutoff = 0.4)
> write.graph(my.network$gR, file = "myNetwork.gml", format = "gml")
```

`plotLoadings` visualises the loading weights of each selected variables on each component and each data set. The color indicates the class in which the variable has the maximum level of expression (`contrib = 'max'`) or minimum (`contrib = 'min'`), on average (`method = 'mean'`) or using the median (`method = 'median'`).

```
> plotLoadings(sgccda.res, comp = 2, contrib = 'max', method = 'median')
```

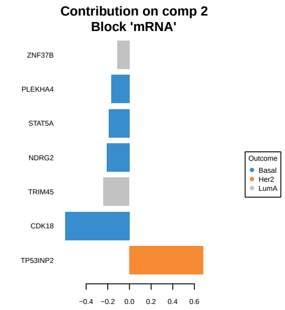

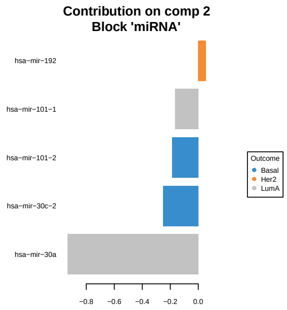

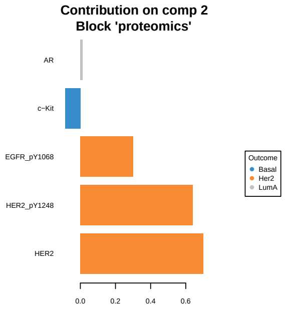

The `cimDiablo` function is a clustered image map specifically implemented to represent the multi-'omics molecular signature expression for each sample.

```
> cimDiablo(sgccda.res, color.blocks = c('darkorchid', 'brown1', 'lightgreen'),
+           comp = 1, margin=c(8,20), legend.position = "right")
```

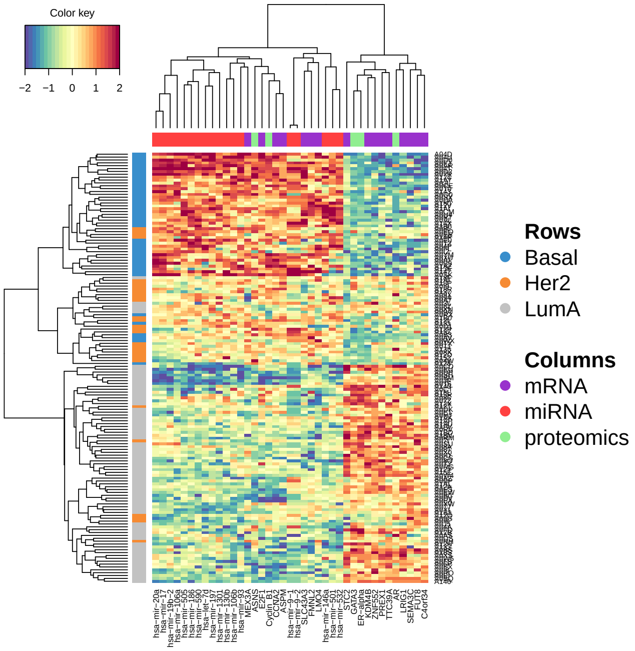

E2.6. Performance of the model

We assess the performance of the model using 10-fold cross-validation repeated 10 times with the function `perf`. The method runs a `block.splsda` model on the pre-specified arguments input from our `sgccda.res` object but on cross-validated samples. We then assess the accuracy of the prediction on the left out samples.

```
> set.seed(123) # for reproducibility, only when the `cpus' argument is not used
> t1 = proc.time()
> perf.diablo = perf(sgccda.res, validation = 'Mfold', folds = 10, nrepeat = 10,
+                    dist = 'centroids.dist')
> t2 = proc.time()
> running_time = t2 - t1; running_time
```

```
  user  system elapsed
24.035   0.530  25.231
```

```
> #perf.diablo  # lists the different outputs
>
> # Performance with Majority vote
> perf.diablo$MajorityVote.error.rate
```

```
$centroids.dist
                comp 1     comp 2
Basal       0.02444444 0.04666667
Her2        0.20666667 0.15666667
LumA        0.04666667 0.01733333
Overall.ER  0.07200000 0.05400000
Overall.BER 0.09259259 0.07355556
```

```
> # Performance with Weighted vote
> perf.diablo$WeightedVote.error.rate
```

```
$centroids.dist
                comp 1     comp 2
Basal       0.01777778 0.04000000
Her2        0.13666667 0.13000000
LumA        0.04666667 0.01733333
Overall.ER  0.05600000 0.04666667
Overall.BER 0.06703704 0.06244444
```

In addition to the usual (balanced) classification error rates, predicted dummy variables and variates, as well as stability of the selected features, the `perf` function for DIABLO outputs the performance based on Majority Vote (each data set votes for a class for a particular test sample) or a weighted vote, where the weight is defined according to the correlation between the latent component associated to a particular data set and the outcome.

Since the `tune` function was used with the `centroid.dist` argument, we examine the outputs of the `perf` function for that same distance.

An AUC plot per block is plotted using the function `auroc`, refer to Rohart et al. 2017 for the interpretation of such output as the ROC and AUC criteria are not particularly insightful in relation to the performance evaluation of our methods, but can complement the statistical analysis.

```
> auc.diablo = auroc(sgccda.res, roc.block = "miRNA", roc.comp = 2)
```

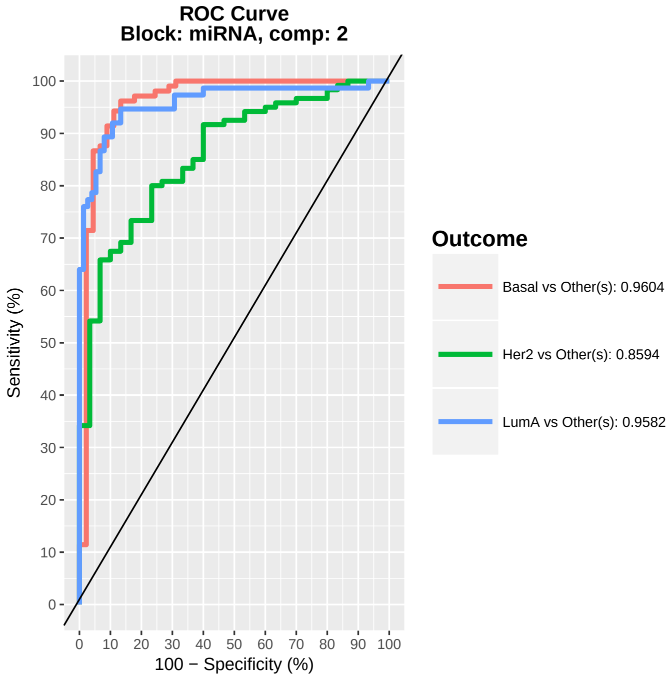

E2.7. Prediction on an external test set.

The `predict` function predicts the class of samples from a test set. In our specific case, one data set is missing in the test set but the method can still be applied. Make sure the name of the blocks correspond exactly.

```
> # prepare test set data: here one block (proteins) is missing
> data.test.TCGA = list(mRNA = breast.TCGA$data.test$mrna,
+                       miRNA = breast.TCGA$data.test$mirna)
> predict.diablo = predict(sgccda.res, newdata = data.test.TCGA)
> # the warning message will inform us that one block is missing
> #predict.diablo # list the different outputs
```

The confusion table compares the real subtypes with the predicted subtypes for a 2 component model, for the distance of interest:

```
> confusion.mat = get.confusion_matrix(truth = breast.TCGA$data.test$subtype,
+                   predicted = predict.diablo$WeightedVote$centroids.dist[,2])
> confusion.mat
```

```
      predicted.as.Basal predicted.as.Her2 predicted.as.LumA
Basal                 20                 1                 0
Her2                   0                14                 0
LumA                   0                 1                34
```

```
> get.BER(confusion.mat)
```

```
[1] 0.02539683
```

E2.8. Session information of this Sweave code

```
> sessionInfo()
```

```
R version 3.4.1 (2017-06-30)
Platform: x86_64-apple-darwin15.6.0 (64-bit)
Running under: macOS Sierra 10.12.6

Matrix products: default
BLAS: /Library/Frameworks/R.framework/Versions/3.4/Resources/lib/libRblas.0.dylib
LAPACK: /Library/Frameworks/R.framework/Versions/3.4/Resources/lib/libRlapack.dylib

locale:
[1] en_AU.UTF-8/en_AU.UTF-8/en_AU.UTF-8/C/en_AU.UTF-8/en_AU.UTF-8

attached base packages:
[1] stats     graphics  grDevices utils     datasets  methods   base

other attached packages:
[1] mixOmics_6.3.0  ggplot2_2.2.1   lattice_0.20-35 MASS_7.3-47

loaded via a namespace (and not attached):
 [1] Rcpp_0.12.13       RSpectra_0.12-0    compiler_3.4.1     RColorBrewer_1.1-2
 [5] plyr_1.8.4         bindr_0.1          tools_3.4.1        digest_0.6.12
 [9] jsonlite_1.5       tibble_1.3.4       gtable_0.2.0       pkgconfig_2.0.1
[13] rlang_0.1.2        Matrix_1.2-11      igraph_1.1.2       shiny_1.0.5
[17] parallel_3.4.1     bindrcpp_0.2       gridExtra_2.3      stringr_1.2.0
[21] dplyr_0.7.4        knitr_1.17         htmlwidgets_0.9    tidyselect_0.2.0
[25] grid_3.4.1         glue_1.1.1         ellipse_0.3-8      R6_2.2.2
[29] rARPACK_0.11-0     rgl_0.98.1         tidyr_0.7.1        purrr_0.2.3
[33] reshape2_1.4.2     corpcor_1.6.9      magrittr_1.5       scales_0.5.0
[37] htmltools_0.3.6    matrixStats_0.52.2 assertthat_0.2.0   mime_0.5
[41] colorspace_1.3-2   xtable_1.8-2       httpuv_1.3.5       labeling_0.3
[45] stringi_1.1.5      lazyeval_0.2.0     munsell_0.4.3
```

```
Writing to file DIABLO-analysis.R
```

References

Therese Sørlie, Charles M Perou, Robert Tibshirani, Turid Aas, Stephanie Geisler, Hilde Johnsen, Trevor Hastie, Michael B Eisen, Matt Van De Rijn, Stefanie S Jeffrey, et al. Gene expression patterns of breast carcinomas distinguish tumor subclasses with clinical implications. *Proceedings of the National Academy of Sciences*, 98(19):10869–10874, 2001.

Cancer Genome Atlas Network et al. Comprehensive molecular portraits of human breast tumours. *Nature*, 490(7418):61–70, 2012.

Amrit Singh, Benoit Gautier, Casey P Shannon, Michael Vacher, Florian Rohart, Scott J Tebutt, and Kim-Anh Lê Cao. Diablo-an integrative, multi-omics, multivariate method for multi-group classification. *bioRxiv*, 067611, 2016.

Florian Rohart, Aida Eslami, Nicholas Matigian, Stephanie Bougeard, and Kim-Anh Lê Cao. Mint: A multivariate integrative approach to identify a reproducible biomarker signature across multiple experiments and platforms. *BMC Bioinformatics*, 18(128), 2017.

Ignacio González, Kim-Anh Lê Cao, Melissa J Davis, Sébastien Déjean, et al. Visualising associations between paired 'omics' data sets. *BioData mining*, 5(1):19, 2012.
