# Supplementary material for: mixOmics: An R package for ‘omics feature selection and multiple data integration
Source: PLoS Comput Biol. 2017 Nov 3;13(11):e1005752. doi: 10.1371/journal.pcbi.1005752 (PMC5687754; doi:10.1371/journal.pcbi.1005752)
Supplement: S1 Appendix — (ZIP) [file pcbi.1005752.s002.zip › mixOmicsRscripts/MINT-analysis.pdf]

E3. P-integration across independent studies with MINT

Contents

E3 P-integration across independent studies with MINT 1
E3.1 Data . . . 1
E3.2 MINT PLS-DA . . . 2
E3.3 MINT sparse PLS-DA . . . 5
E3.3.1 Choice of parameters . . . 5
E3.3.2 Final MINT sPLS-DA model . . . 6
E3.3.3 Sample plots . . . 7
E3.3.4 CIM . . . 9
E3.3.5 Loading plots . . . 9
E3.3.6 Performance assessment and prediction . . . 10
E3.4 Session information of this Sweave code . . . 12

We first load mixOmics:

```
> library(mixOmics)
```

E3.1. Data

In our original study we integrated 15 transcriptomics microarray stem cells datasets to classify three types of human cells: human Fibroblasts (Fib), human Embryonic Stem Cells (hESC) and human induced Pluripotent Stem Cells (hiPSC). Here we illustrate only a subset of those data, including 4 transcriptomics studies (125 samples in total) and the levels of expression of 400 transcripts. The data were obtained from the Stemformatics database (www.stemformatics.org, Wells et al. 2013). There exists a biological hierarchy among the three cell types. On the one hand, differences between pluripotent (hiPSC and hESC) and non-pluripotent cells (Fib) are well-characterised and are expected to contribute to the main biological variation. On the other hand, hiPSC are genetically reprogrammed to behave like hESC and both cell types are commonly assumed to be alike. However, differences have been reported in the literature Bilic and Belmonte (2012); Chin et al. (2009); Newman and Cooper (2010). The MINT analysis presented below addresses both subclassification problems in a single analysis.

We first load the data from mixOmics and set up the categorical outcome y and the study membership:

```
> data(stemcells)
> #the combined data set X
> X = stemcells$gene
> dim(X)
```

```
[1] 125 400
```

```
> # the outcome vector Y:
> Y = stemcells$celltype
> length(Y)
```

```
[1] 125
```

```
> summary(Y)
```

```
Fibroblast       hESC       hiPS
        30         37         58
```

```
> # the vector indicating each independent study
> study = stemcells$study
> # number of samples per study:
> summary(study)
```

```
 1  2  3  4
38 51 21 15
```

```
> # experimental design
> table(Y,study)
```

```
            study
Y             1  2  3  4
  Fibroblast  6 18  3  3
  hESC       20  3  8  6
  hiPS       12 30 10  6
```

E3.2. MINT PLS-DA

We first perform a MINT PLS-DA with all variables included in the model and `ncomp = 5` components. The `perf` function is used to estimate the performance of the model and choose the optimal number of components in `$choice.ncomp` for our final model.

Elapsed running time is reported in seconds.

```
> mint.plsda.res.perf = mint.plsda(X = X, Y = Y, study = study, ncomp = 5)
> set.seed(2543)  # for reproducible result in this example
> t1 = proc.time()
> perf.mint.plsda.cell <- perf(mint.plsda.res.perf, validation = "Mfold", folds = 5,
+                    progressBar = FALSE, auc = TRUE)
> t2 = proc.time()
> running_time = t2 - t1; running_time
```

```
   user  system elapsed
  2.664   0.047   2.793
```

```
> plot(perf.mint.plsda.cell, col = color.mixo(5:7))
```

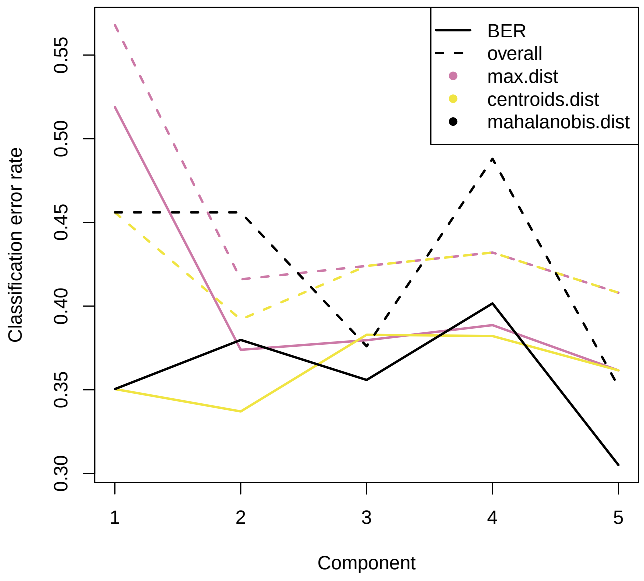

Based on the performance plot above, `ncomp = 1` seems the achieve the best performance for both maximum and centroids distances in terms of BER (see supplemental information from Rohart et al. 2017 for more details about the prediction distances).

Additional numerical outputs such as the BER and overall error rates per component, and the error rates per class and per prediction distance can be output.

```
> perf.mint.plsda.cell$global.error
```

```
$BER
        max.dist centroids.dist mahalanobis.dist
comp 1 0.5189500      0.3504194        0.3504194
comp 2 0.3738739      0.3370612        0.3797763
comp 3 0.3796210      0.3828829        0.3558662
comp 4 0.3886300      0.3821062        0.4016051
comp 5 0.3616030      0.3616030        0.3050637

$overall
       max.dist centroids.dist mahalanobis.dist
comp 1    0.568          0.456            0.456
comp 2    0.416          0.392            0.456
comp 3    0.424          0.424            0.376
comp 4    0.432          0.432            0.488
comp 5    0.408          0.408            0.352

$error.rate.class
$error.rate.class$max.dist
              comp 1    comp 2    comp 3    comp 4    comp 5
Fibroblast 0.0000000 0.0000000 0.0000000 0.0000000 0.0000000
hESC       0.9189189 0.6216216 0.6216216 0.6486486 0.5675676
hiPS       0.6379310 0.5000000 0.5172414 0.5172414 0.5172414
```

```
$error.rate.class$centroids.dist
              comp 1    comp 2    comp 3    comp 4    comp 5
Fibroblast 0.0000000 0.0000000 0.0000000 0.0000000 0.0000000
hESC       0.1891892 0.4594595 0.6486486 0.5945946 0.5675676
hiPS       0.8620690 0.5517241 0.5000000 0.5517241 0.5172414

$error.rate.class$mahalanobis.dist
              comp 1    comp 2     comp 3     comp 4    comp 5
Fibroblast 0.0000000 0.0000000 0.06666667 0.03333333 0.0000000
hESC       0.1891892 0.4324324 0.62162162 0.37837838 0.4324324
hiPS       0.8620690 0.7068966 0.37931034 0.79310345 0.4827586
```

The optimal number of components:

```
> perf.mint.plsda.cell$choice.ncomp
```

```
        max.dist centroids.dist mahalanobis.dist
overall        1              1                1
BER            1              1                1
```

Despite having to choose 1 component, we run a final MINT PLS-DA model for `ncomp = 2` in order to obtain 2D graphical outputs.

```
> mint.plsda.res = mint.plsda(X = X, Y = Y, study = study, ncomp = 2)
> #mint.plsda.res # lists the different functions
> plotIndiv(mint.plsda.res, legend = TRUE, title = 'MINT PLS-DA',
+            subtitle = 'stem cell study', ellipse = T)
```

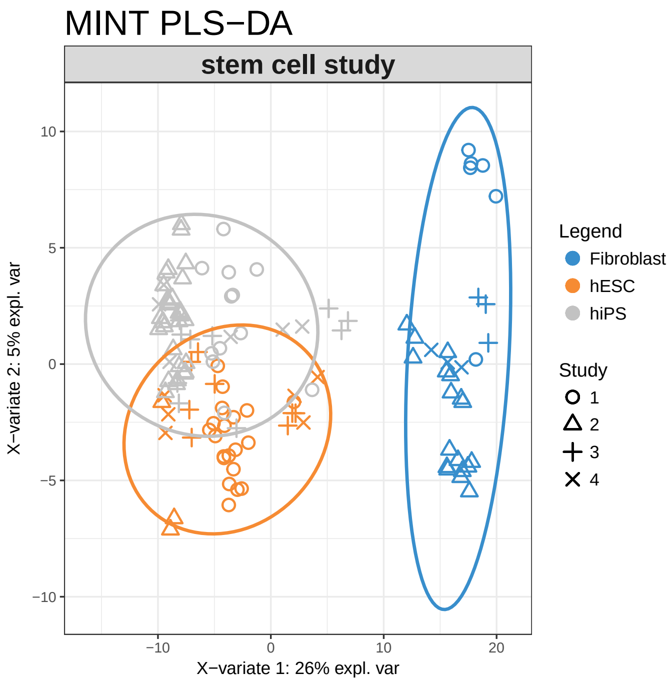

The sample plot shows that fibroblasts are separated on the first MINT PLS-DA component. We observe that while deemed not crucial for an optimal discrimination, the second component seems to discriminate hESC and hiPSC.

E3.3. MINT sparse PLS-DA

The MINT PLS-DA model is built on all 400 genes in X, many of which may be uninformative to characterise the different classes. The MINT sPLS-DA analysis aims to identify a small subset of genes that best discriminate the classes.

E3.3.1 Choice of parameters

We first tune the `keepX` parameter using the `tune` function for a MINT object. The function performs Leave-One-Group-Out-Cross-Validation (LOGOCV) for different values of `test.keepX` provided on each component, therefore no repeated CV is needed. Based on the mean classification error rate (overall error rate or BER), it will output the optimal number of component `ncomp` and the optimal `keepX` to be included in the final model.

```
> t1 = proc.time()
> tune.mint = tune(X = X, Y = Y, study = study, ncomp = 2, test.keepX = seq(1, 100, 1),
+ method = 'mint.splsda', dist = "max.dist", progressBar = FALSE)
> t2 = proc.time()
> # tune.mint   # lists the different types of outputs
>
> # mean error rate per component and per tested keepX value
> # tune.mint$error.rate
>
> running_time = t2 - t1; running_time
```

```
   user  system elapsed 
 28.788   1.537  30.612 
```

```
> # optimal number of components
> tune.mint$choice.ncomp #tune.mint$choice.ncomp # tell us again than ncomp=1 is sufficient
```

```
$ncomp
[1] 1

$values
     comp 1    comp 2
1 0.3888889 0.4555556
2 0.3333333 0.2666667
3 0.4333333 0.4916667
4 0.3888889 0.2222222
```

```
> # optimal keepX
> tune.mint$choice.keepX
```

```
comp 1 comp 2 
     6     16 
```

```
> plot(tune.mint, col = color.jet(2))
```

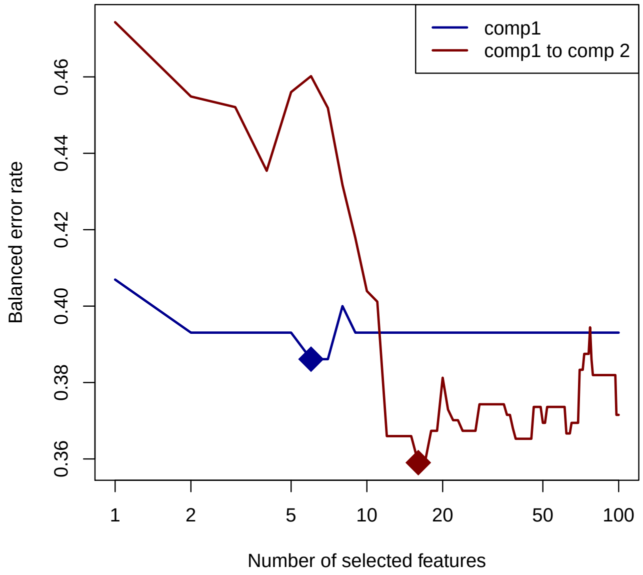

E3.3.2 Final MINT sPLS-DA model

Following the tuning results, our final MINT sPLS-DA model is as follows. Note that we still chose a model with 2 components in order to obtain 2D graphics.

```
> mint.splsda.res = mint.splsda(X = X, Y = Y, study = study, ncomp = 2,
+                               keepX = tune.mint$choice.keepX)
>
> #mint.splsda.res   # lists useful functions that can be used with a MINT object
```

The `selectVar` function outputs the selected transcripts on the first component along with their loading weight value. We consider variables as important in the model when their absolute loading weight value is high. In addition to this output, we could compare the stability of the selected features across studies (see Example in the PLS-DA analysis in Rohart et al. 2017).

```
> selectVar(mint.splsda.res, comp = 1)
```

```
$name
[1] "ENSG00000181449" "ENSG00000123080" "ENSG00000110721" "ENSG00000176485"
[5] "ENSG00000184697" "ENSG00000102935"

$value
                  value.var
ENSG00000181449 -0.70533253
ENSG00000123080  0.48230598
ENSG00000110721 -0.44201007
ENSG00000176485 -0.23247562
```

```
ENSG00000184697 -0.10494626
ENSG00000102935 -0.09723903

$comp
[1] 1
```

E3.3.3 Sample plots

The samples can be projected on the global components:

```
> plotIndiv(mint.splsda.res, study = 'global', legend = TRUE, title = 'MINT sPLS-DA',
+           subtitle = 'Global', ellipse=T)
```

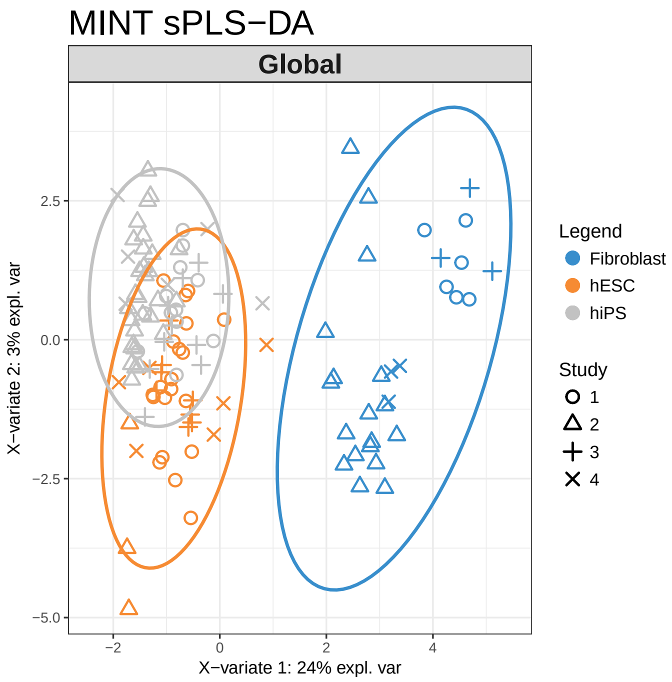

Or, alternatively using the MINT PLS-component from each study. This options allows us to examine each study individually.

```
> plotIndiv(mint.splsda.res, study = 'all.partial',  title = 'MINT sPLS-DA',
+           subtitle = paste("Study",1:4))
```

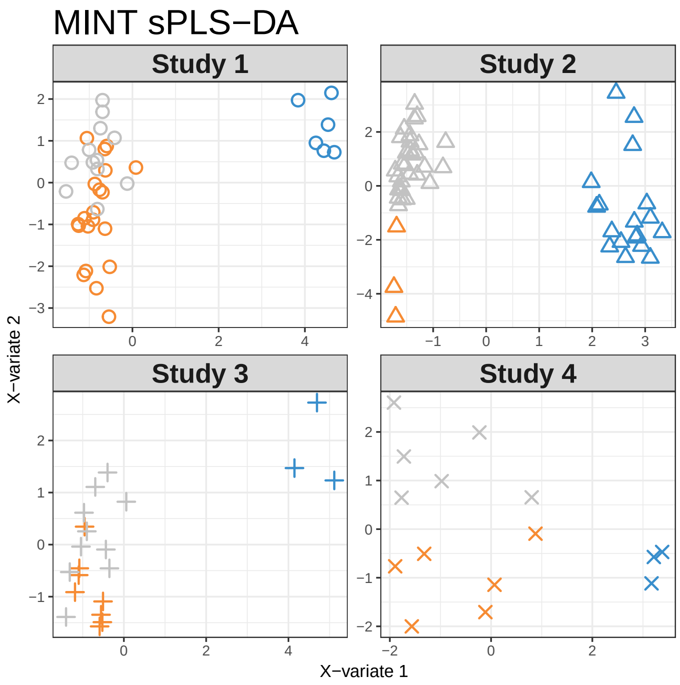

Additionally, the `plotArrow` function displays each sample from the X-components (start of the arrow) to the Y-components (end of the arrow):

```
> plotArrow(mint.splsda.res)
```

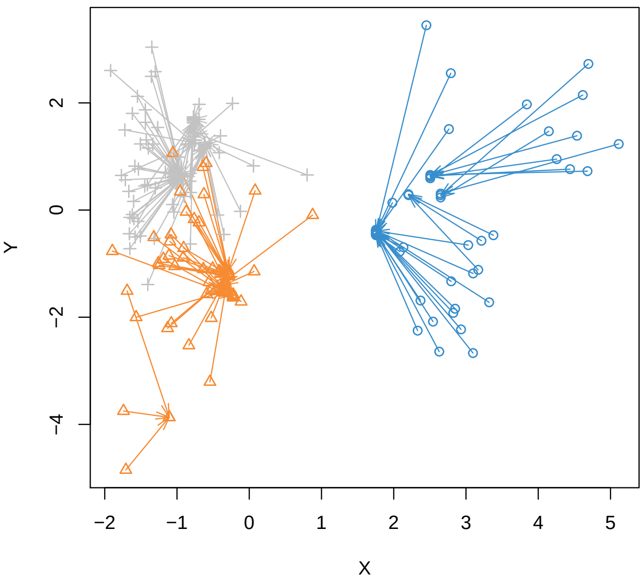

E3.3.4 CIM

A Clustered Image Map including the final gene signature is plotted (default values to Euclidian distance and Complete linkage). Specific component can be also be chosen.

```
> cim(mint.splsda.res, comp = 1, margins=c(10,5),
+     row.sideColors = color.mixo(as.numeric(Y)), row.names = FALSE,
+     title = "MINT sPLS-DA, component 1")
```

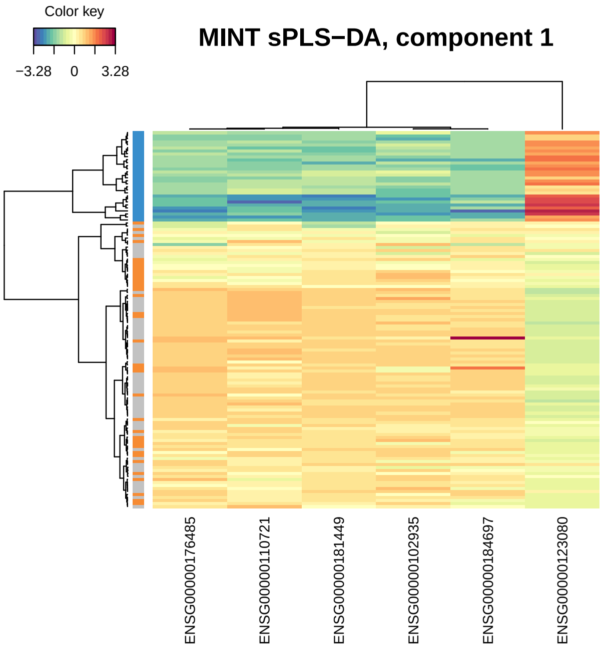

E3.3.5 Loading plots

Finally the `plotLoadings` function displays the coefficient weight of each selected variable in each study and shows the agreement of the gene signature across studies. Colors indicate the class in which the mean expression value of each selected gene is maximal. For component 1:

```
> plotLoadings(mint.splsda.res, contrib="max", method = 'mean', comp=1,
+              study="all.partial", legend=FALSE, title="Contribution on comp 1",
+              subtitle = paste("Study",1:4))
```

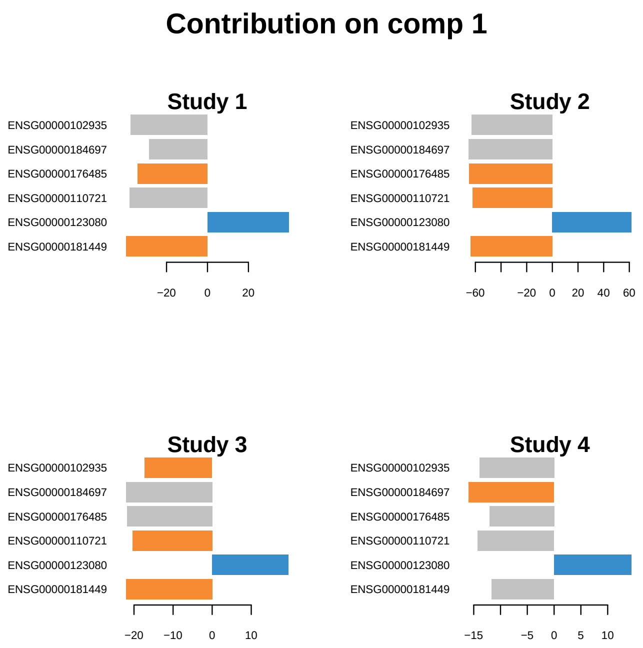

Other visualisation functions include `plotVar`.

E3.3.6 Performance assessment and prediction

The performance of the MINT sPLS-DA model can be assessed with the `perf` function. Since the previous tuning of the MINT sPLS-DA model was conducted with the distance `max.dist`, the same distance is used to assess the performance of the final model.

```
> set.seed(123)
> t1 = proc.time()
> perf.mint = perf(mint.splsda.res, progressBar = FALSE, dist = 'max.dist')
> t2 = proc.time()
> running_time = t2 - t1; running_time
```

```
   user  system elapsed
  0.231   0.014   0.255
```

```
> perf.mint$global.error
```

```
$BER
        max.dist
comp 1 0.5132029
comp 2 0.3918919

$overall
       max.dist
comp 1    0.560
comp 2    0.432

$error.rate.class
```

```
$error.rate.class$max.dist
              comp 1    comp 2
Fibroblast 0.0000000 0.0000000
hESC       0.9189189 0.6756757
hiPS       0.6206897 0.5000000
```

```
> plot(perf.mint, col = color.mixo(5))
```

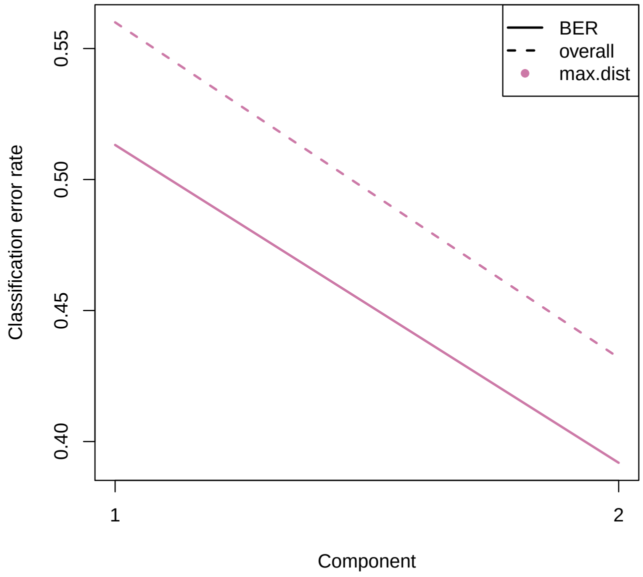

A `predict` function is also available to predict the class membership of new test samples from an external study.

```
> # we predict on study 3
> ind.test = which(study == "3")
> test.predict <- predict(mint.splsda.res, newdata = X[ind.test, ], dist = "max.dist",
+                         study.test = factor(study[ind.test]))
> Prediction <- test.predict$class$max.dist[, 2]
> # the confusion table compares the real subtypes with the predicted subtypes
> get.confusion_matrix(truth = Y[ind.test],
+                      predicted = Prediction)
```

```
           predicted.as.Fibroblast predicted.as.hESC predicted.as.hiPS
Fibroblast                       3                 0                 0
hESC                             0                 6                 2
hiPS                             0                 3                 7
```

An AUC plot for the integrated data can be obtained using the function `auroc`.

```
> auc.mint.splsda = auroc(mint.splsda.res, roc.comp = 2)
```

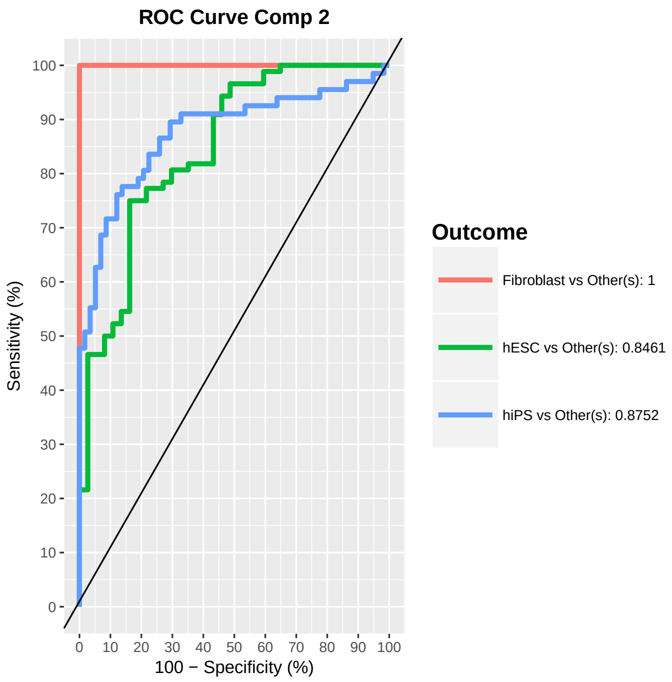

We can also obtain an AUC plot per study.

```
> auc.mint.splsda = auroc(mint.splsda.res, roc.comp = 2, roc.study = '2')
```

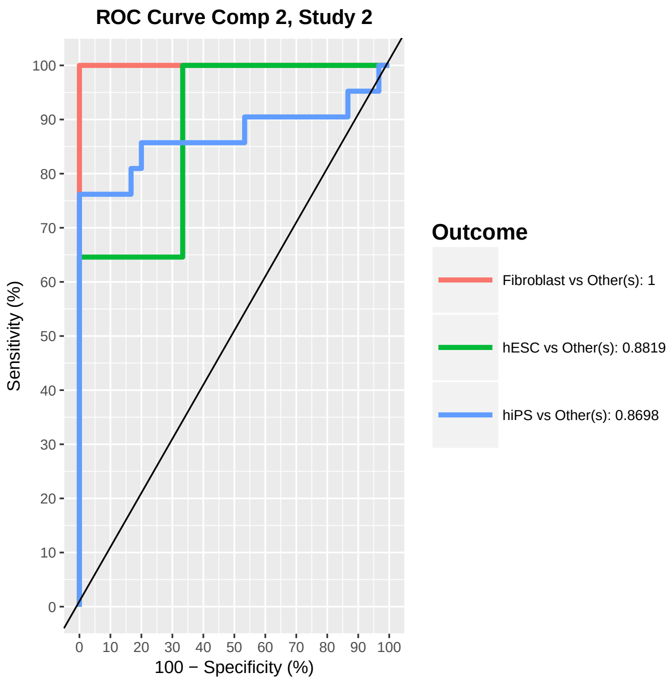

E3.4. Session information of this Sweave code

```
> sessionInfo()
```

```
R version 3.4.1 (2017-06-30)
Platform: x86_64-apple-darwin15.6.0 (64-bit)
Running under: macOS Sierra 10.12.6

Matrix products: default
BLAS: /Library/Frameworks/R.framework/Versions/3.4/Resources/lib/libRblas.0.dylib
LAPACK: /Library/Frameworks/R.framework/Versions/3.4/Resources/lib/libRlapack.dylib

locale:
[1] en_AU.UTF-8/en_AU.UTF-8/en_AU.UTF-8/C/en_AU.UTF-8/en_AU.UTF-8

attached base packages:
[1] stats     graphics  grDevices utils     datasets  methods   base

other attached packages:
[1] mixOmics_6.3.0  ggplot2_2.2.1   lattice_0.20-35 MASS_7.3-47

loaded via a namespace (and not attached):
 [1] Rcpp_0.12.13       RSpectra_0.12-0    compiler_3.4.1     RColorBrewer_1.1-2
 [5] plyr_1.8.4         bindr_0.1          tools_3.4.1        digest_0.6.12
 [9] jsonlite_1.5       tibble_1.3.4       gtable_0.2.0       pkgconfig_2.0.1
[13] rlang_0.1.2        Matrix_1.2-11      igraph_1.1.2       shiny_1.0.5
[17] parallel_3.4.1     bindrcpp_0.2       gridExtra_2.3      stringr_1.2.0
[21] dplyr_0.7.4        knitr_1.17         htmlwidgets_0.9    grid_3.4.1
[25] glue_1.1.1         ellipse_0.3-8      R6_2.2.2           rARPACK_0.11-0
[29] rgl_0.98.1         tidyr_0.7.1        purrr_0.2.3        reshape2_1.4.2
[33] corpcor_1.6.9      magrittr_1.5       scales_0.5.0       htmltools_0.3.6
[37] matrixStats_0.52.2 assertthat_0.2.0   mime_0.5           colorspace_1.3-2
[41] xtable_1.8-2       httpuv_1.3.5       labeling_0.3       stringi_1.1.5
[45] lazyeval_0.2.0     munsell_0.4.3
```

```
Writing to file MINT-analysis.R
```

References

Christine A Wells, Rowland Mosbergen, Othmar Korn, Jarny Choi, Nick Seidenman, Nicholas A Matigian, Alejandra M Vitale, and Jill Shepherd. Stemformatics: visualisation and sharing of stem cell gene expression. *Stem Cell Res.*, 10(3):387–395, 2013.

Josipa Bilic and Juan Carlos Izpisua Belmonte. Concise review: Induced pluripotent stem cells versus embryonic stem cells: close enough or yet too far apart? *Stem Cells*, 30(1):33–41, 2012.

Mark H Chin, Mike J Mason, Wei Xie, Stefano Volinia, Mike Singer, Cory Peterson, Gayane Ambartsumyan, Otaren Aimiuwu, Laura Richter, Jin Zhang, et al. Induced pluripotent stem cells and embryonic stem cells are distinguished by gene expression signatures. *Cell stem cell*, 5(1): 111–123, 2009.

Aaron M Newman and James B Cooper. Lab-specific gene expression signatures in pluripotent stem cells. *Cell stem cell*, 7(2):258–262, 2010.

Florian Rohart, Benoit Gautier, Amrit Singh, and Kim-Anh Lê Cao. mixomics: an r package for 'omics feature selection and multiple data integration. *bioRxiv*, 108597, 2017.
