# Supplementary material for: mixOmics: An R package for ‘omics feature selection and multiple data integration
Source: PLoS Comput Biol. 2017 Nov 3;13(11):e1005752. doi: 10.1371/journal.pcbi.1005752 (PMC5687754; doi:10.1371/journal.pcbi.1005752)
Supplement: S1 Appendix — (ZIP) [file pcbi.1005752.s002.zip › mixOmicsRscripts/PLSDA-analysis.pdf]

E1. Single ‘omics supervised multivariate analyses

Contents

E1 Single ‘omics supervised multivariate analyses **1**
E1.1 Data 1
E1.2 Principal Component Analysis 1
E1.3 PLS-DA analysis 3
E1.4 sPLS-DA analysis 7
E1.5 Other graphical outputs 13
E1.6 Other outputs 18
E1.7 Session information of this Sweave code 18

We first load mixOmics:

```
> library(mixOmics)
```

E1.1. Data

The data are directly available in a processed and normalised format from the package. The Small Round Blue Cell Tumors (SRBCT) dataset from Khan et al. (2001) includes the expression levels of 2,308 genes measured on 63 samples. The samples are classified into four classes as follows: 8 Burkitt Lymphoma (BL), 23 Ewing Sarcoma (EWS), 12 neuroblastoma (NB), and 20 rhabdomyosarcoma (RMS).

```
> data(srbct)
> X = srbct$gene  #the gene expression data
> dim(X)
```

```
[1]   63 2308
```

```
> summary(srbct$class)
```

```
EWS  BL  NB RMS
 23   8  12  20
```

E1.2. Principal Component Analysis

A preliminary PCA analysis on the gene expression data allows a first exploration of the major sources of variation in the data. Recall that PCA is an unsupervised analysis where no information about the tumour classes is provided in the method. In order to understand the amount of variation explained, we set `ncomp` to a rather large number. In PCA, centering is a recommended transformation in most situations Wold et al. (2001) and results in all genes with the same zero mean, allowing to focus on the differences between samples. Scaling generally aims to give similar weights to all genes in the analysis, since genes with high variance will be considered influential in PCA but are not necessarily of biological relevance.

```
> pca.srbct = pca(X, ncomp = 10, center = TRUE, scale = TRUE)
> #pca.srbct #outputs the explained variance per component
> plot(pca.srbct)  # screeplot of the eingenvalues (explained variance per component)
```

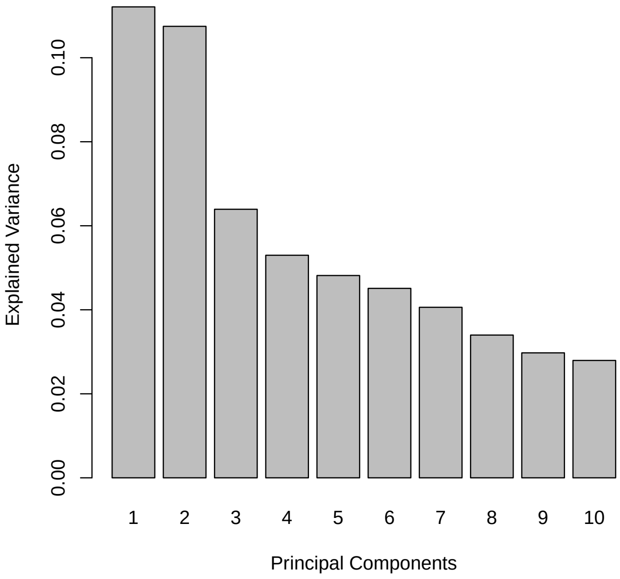

The barplot above shows that two components are sufficient to explain most variance (or information) from the data.

In the following sample plot, samples are represented on the first two principal components and colored according to the tumour type. Here we observe that the major source of variation may not be explained by tumour types. Note that since PCA is unsupervised, we only take into account the sample type information *after* the PCA, for visualisation purposes.

```
> plotIndiv(pca.srbct, group = srbct$class, ind.names = FALSE,
+           legend = TRUE, title = 'PCA on SRBCT')
```

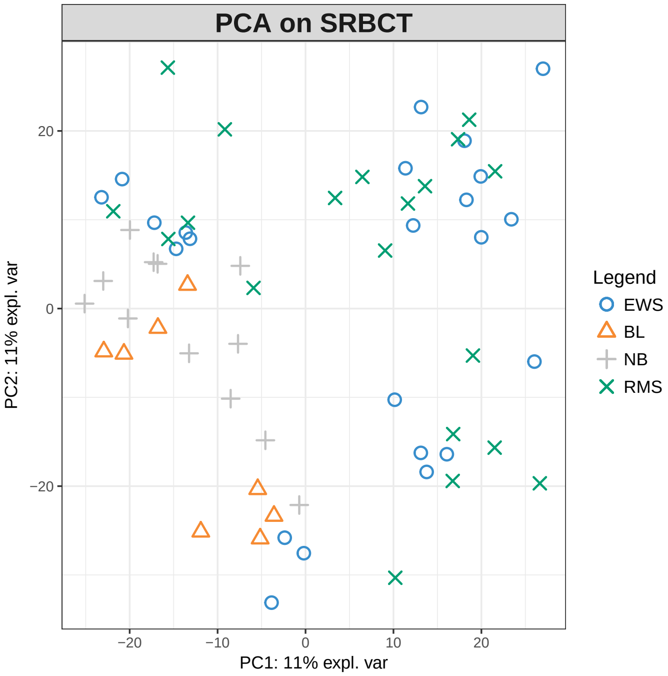

E1.3. PLS-DA analysis

For a discriminant analysis, we set the factor Y that indicates the class membership of each sample. Inside the PLS-DA procedure the Y factor will be transformed into a dummy matrix.

```
> Y = srbct$class
> summary(Y)        #outcome categories
```

```
EWS  BL  NB RMS
 23   8  12  20
```

A PLS-DA model is fitted with ten components to evaluate the performance and the number of components necessary for the final model (see below).

Sample plots. The samples are then projected into the subspace spanned by the first two components.

```
> srbct.plsda <- plsda(X, Y, ncomp = 10)  # set ncomp to 10 for performance assessment later
> plotIndiv(srbct.plsda , comp = 1:2,
+           group = srbct$class, ind.names = FALSE,
+           ellipse = TRUE, legend = TRUE, title = 'PLSDA on SRBCT')
```

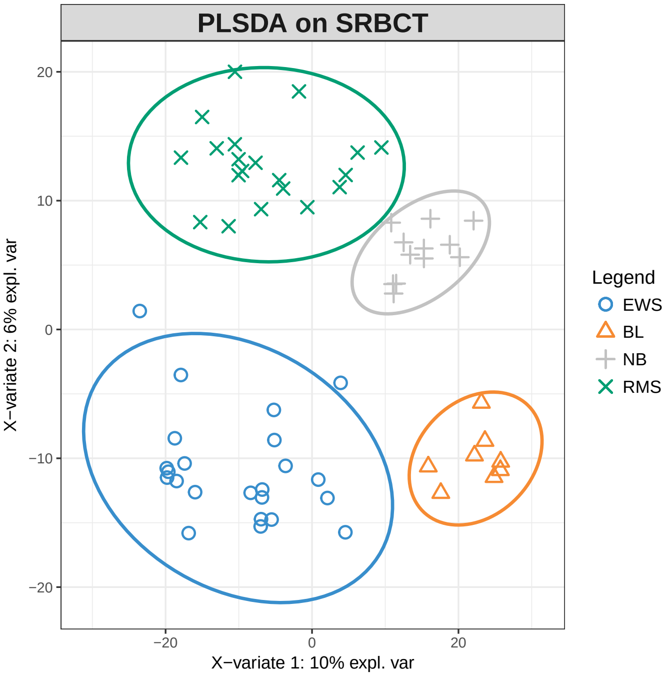

From the sample plot, we observe a clear separation of the four tumor classes compared to an unsupervised PCA sample plot. Confidence ellipses for each class are plotted to highlight the strength of the discrimination (confidence level set to 95% by default, see argument `ellipse.level`).

As detailed in our main article and supplemental information (Rohart et al., 2017) the prediction area can be visualised by calculating a background surface first, before overlaying the sample plot. See `?background.predict` for more details. The prediction distance needs to be specified:

```
> # with background
> background = background.predict(srbct.plsda, comp.predicted=2, dist = "max.dist")
> #optional: xlim = c(-40,40), ylim = c(-30,30))
>
> plotIndiv(srbct.plsda, comp = 1:2,
+           group = srbct$class, ind.names = FALSE, title = "Maximum distance",
+           legend = TRUE,  background = background)
```

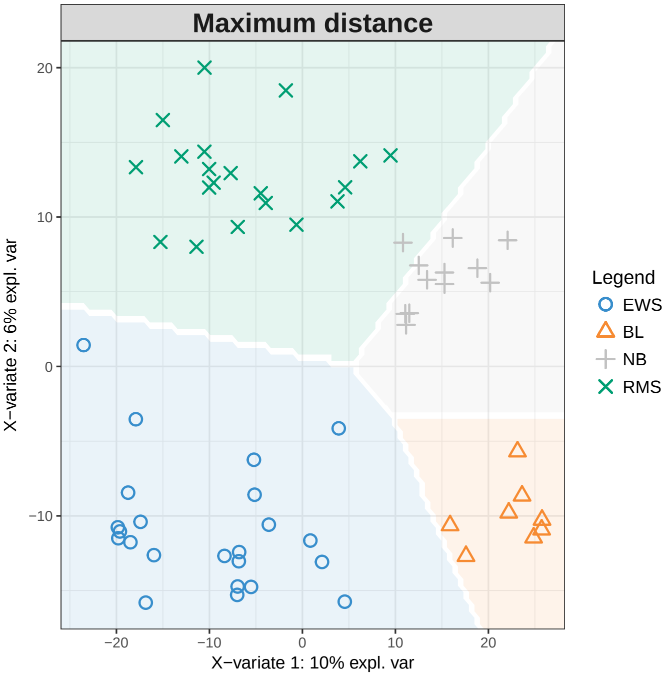

Performance. The classification performance of the PLS-DA model is assessed with the `perf` function using 5-fold cross-validation repeated 10 times. The number of repeats is necessary to ensure a good estimate of the classification error rate (as the CV-folds are determined in a random manner). From the performance results we can decide on the choice of the number of components of the final PLS model. The maximal number of components assessed corresponds to the number of components of the `srbct.plsda` model run above.

We report the elapsed running time (in seconds).

```
> # may take some time to run
> set.seed(2543) # for reproducibility
> t1 = proc.time()
> perf.plsda.srbct <- perf(srbct.plsda, validation = "Mfold", folds = 5,
+                          progressBar = FALSE, auc = TRUE, nrepeat = 10)
> t2 = proc.time()
> running_time = t2 - t1; running_time # running time
```

```
   user  system elapsed
 73.717   3.368  81.578
```

```
> # perf.plsda.srbct$error.rate  # error rates
> plot(perf.plsda.srbct, col = color.mixo(5:7), sd = TRUE, legend.position = "horizontal")
```

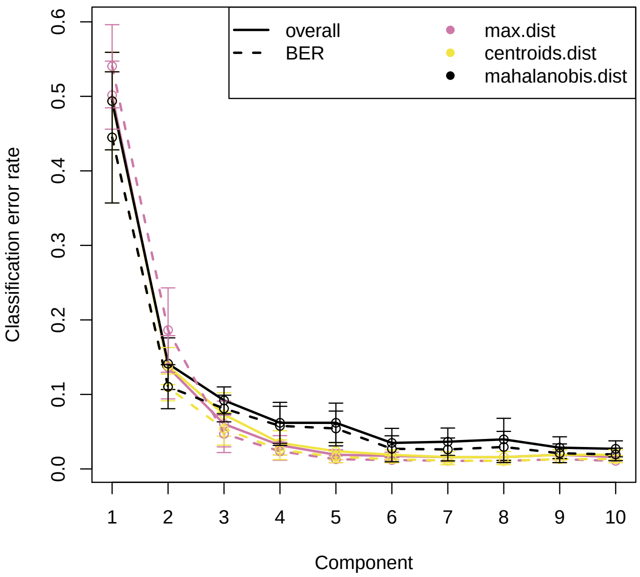

From the performance plot, we observe that the overall error rate and the Balanced Error Rate (BER) are similar, and sharply decrease from 1 to 3 components. The error rates stabilise after 6 components. The *perf* function outputs the optimal number of components in `perf.plsda.srbct$choice.ncomp` based on t-tests that test for a significant difference in the mean error rate between components. The result is output for each performance measure and distance, when applicable. The performance displayed suggests that `ncomp = 6` with BER and the maximum distance is sufficient to achieve good performance (0.06 error rate).

An AUC plot can also be obtained using the function `auroc`, where the AUC is calculated from training cross-validation sets and averaged (see the `perf` function outputs for each component, `perf.plsda.srbct$auc` and `perf.plsda.srbct$auc.all` for one vs. one class or one vs. all classes). Note however that ROC and AUC criteria may not be particularly insightful, or be in agreement with the PLSDA performance, as the prediction threshold in PLS-DA is based on specified distance as described in the main manuscript (Rohart et al., 2017).

```
> auc.plsda = auroc(srbct.plsda, roc.comp = 6)
```

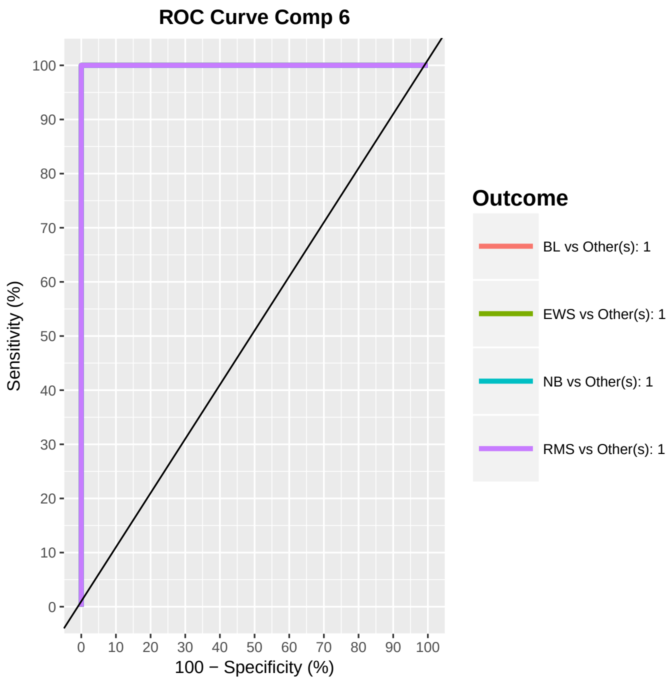

E1.4. sPLS-DA analysis

The PLS-DA model is built on all 2308 genes in X, many of which may be uninformative to characterise the different classes. The sPLS-DA analysis aims to identify a small subset of genes that best discriminate the classes.

Tuning sPLS-DA. We first estimate the classification performance (error rate) with respect to the number of selected variables in the model with the function `tune.splsda`. The tuning is performed one component at a time and we set a maximum of `ncomp = 6` as suggested from the PLS-DA performance assessment. We chose 5-fold cross validation (`folds = 5`) repeated 10 times, and specify a prediction distance (maximal distance) to predict class membership across all CV runs.

The code below may takes a few minutes to run on a mid-range laptop over a grid of `keepX` values that indicate the number of variables to select on each component. Some warnings may appear for some of the runs, as we set a stringent tolerance threshold in the algorithm. The argument `cpus` can be used to run the code in parallel and speed up computational time.

Here we have saved the results into a RData object, so this code is not run during the Sweave compilation. The elapsed time is reported in seconds.

```
> set.seed(2543) # for reproducibility
> # grid of possible keepX values that will be tested for each component
> list.keepX <- c(1:10,  seq(20, 300, 10))
> t1 = proc.time()
> tune.splsda.srbct <- tune.splsda(X, Y, ncomp = 6, validation = 'Mfold', folds = 5,
+                             progressBar = TRUE, dist = 'max.dist', measure = "BER",
+                            test.keepX = list.keepX, nrepeat = 10)
> t2 = proc.time()
> running_time = t2 - t1; running_time # running time
```

```
   user  system elapsed 
512.013  11.576 535.528 
```

```
> error <- tune.splsda.srbct$error.rate  # error rate per component for the keepX grid
```

The number of optimal components to choose is returned in `$choice.ncomp$ncomp`:

```
> ncomp <- tune.splsda.srbct$choice.ncomp$ncomp # optimal number of components based on t-tests
> ncomp
```

```
[1] 3
```

The number of features to select on each component is output in `$choice.keepX`:

```
> select.keepX <- tune.splsda.srbct$choice.keepX[1:ncomp]  # optimal number of variables to select
> select.keepX
```

```
comp1 comp2 comp3 
   10   300    30 
```

The classification error rates for each component conditional on the last component are represented below, for all components specified in the `tune` function.

```
> plot(tune.splsda.srbct, col = color.jet(6))
```

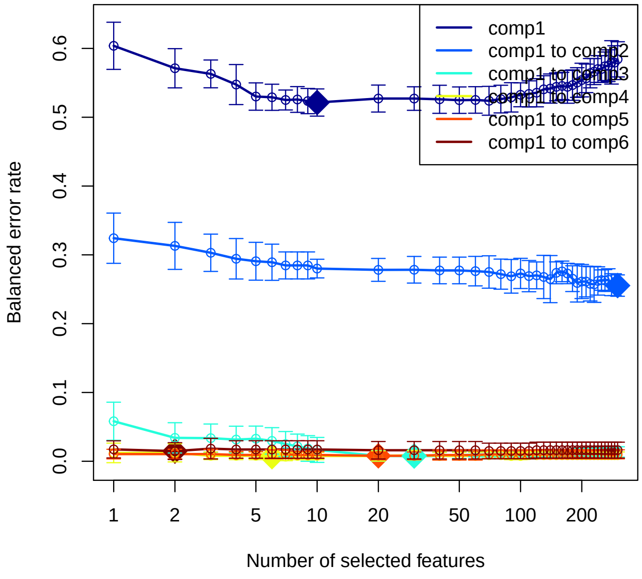

The classification error rate decreases when more components are included in sPLS-DA (the lower the prediction accuracy the better). The optimal number of variables to select that led to the best performance for each component is indicated as a diamond. A number of 3 components is sufficient for our final sPLS-DA model to reach optimal performance.

Final model and sample representation Our final model includes 3 components and 10, 300, 30 selected variables on the first 3 components.

```
> splsda.srbct <- splsda(X, Y, ncomp = ncomp, keepX = select.keepX)
```

The sample plots on the first three components (see below) show that the BL tumours are well separated on the first component, while the second component discriminated EWB from NB and RMS.

```
> plotIndiv(splsda.srbct, comp = c(1,2),
+           group = srbct$class, ind.names = FALSE,
+           ellipse = TRUE, legend = TRUE,
+           title = 'sPLS-DA on SRBCT, comp 1 & 2')
```

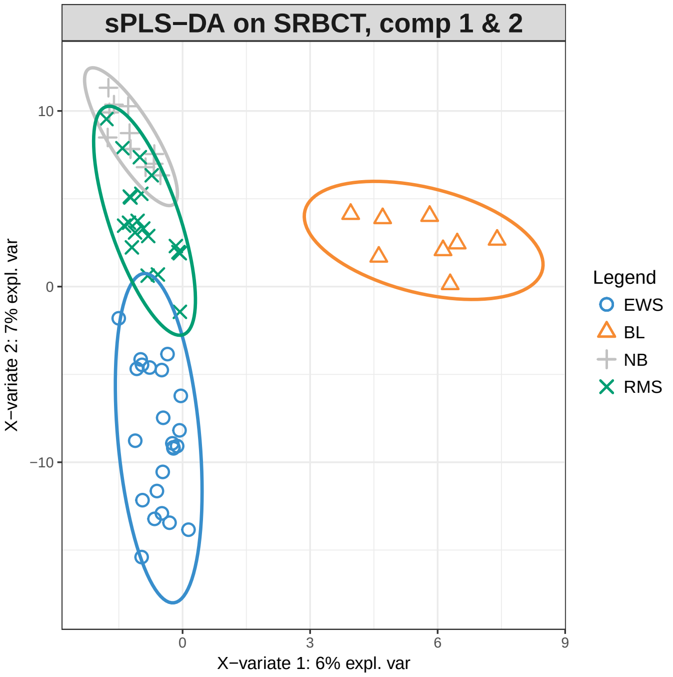

The addition of the third component further discriminates NB from RMS:

```
> plotIndiv(splsda.srbct, comp = c(1,3),
+           group = srbct$class, ind.names = FALSE,
+           ellipse = TRUE, legend = TRUE,
+           title = 'sPLS-DA on SRBCT, comp 1 & 3')
```

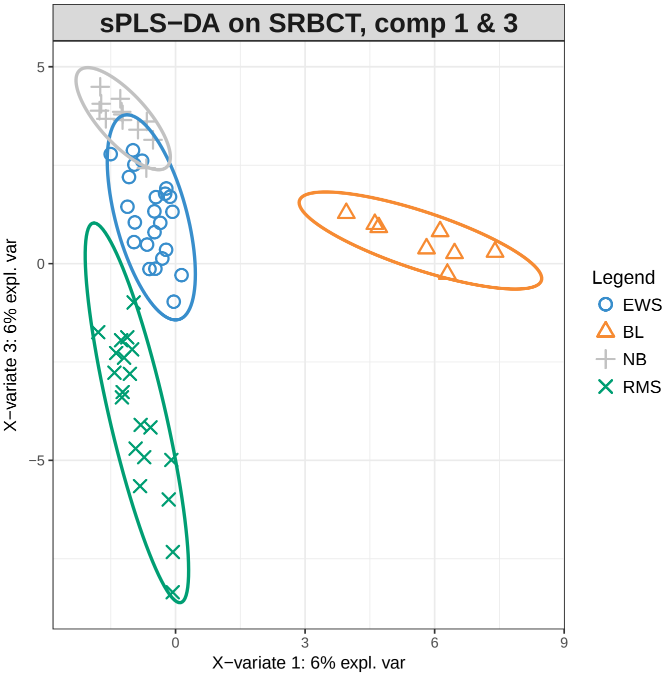

An AUC plot can also be obtained using the function `auroc`, as for the PLS-DA analysis. The first AUROC includes 2 components only:

```
> auc.splsda = auroc(splsda.srbct, roc.comp = 2)
```

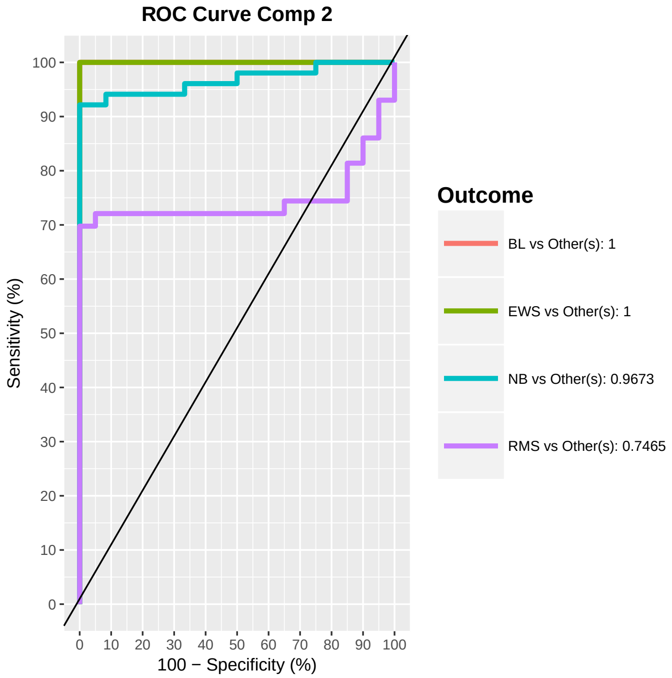

The AUROC including all components from our final model led to a perfect discrimination:

```
> auc.splsda = auroc(splsda.srbct, roc.comp = ncomp)
```

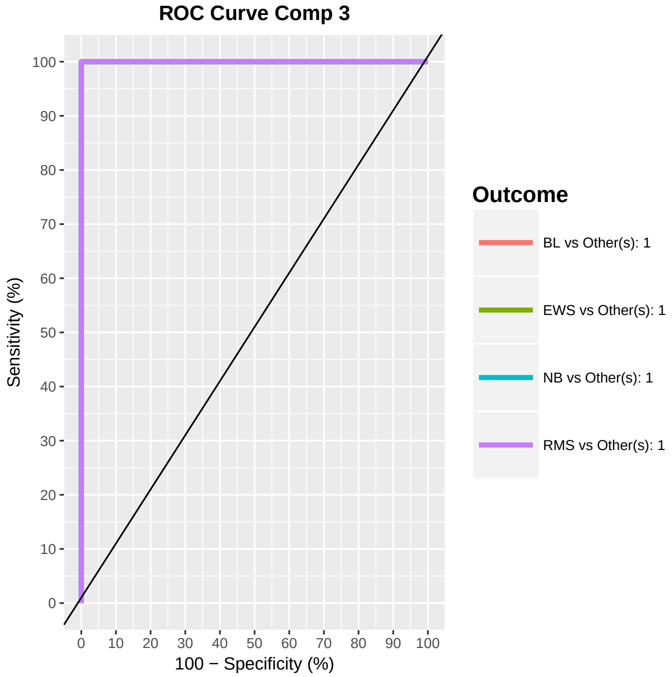

Performance assessment. The classification performance of the *final* sPLS-DA model is assessed with the perf function by specifying a prediction distance. The elapsed running time is reported in seconds.

```
> set.seed(40) # for reproducibility, only when the `cpus' argument is not used
> t1 = proc.time()
> perf.srbct <- perf(splsda.srbct, validation = "Mfold", folds = 5,
+                    dist = 'max.dist', nrepeat = 10,
+                    progressBar = FALSE)
> t2 = proc.time()
> running_time = t2 - t1; running_time
```

```
   user  system elapsed
 29.789   0.571  30.866
```

We observe that the overall and Balanced Error Rate (BER) per class decrease as more components (3) are added in the model.

```
> # perf.srbct  # lists the different outputs
> perf.srbct$error.rate
```

```
$overall
         max.dist
comp 1 0.42857143
comp 2 0.22063492
comp 3 0.00952381
```

```
$BER
          max.dist
comp 1 0.517382246
comp 2 0.245018116
comp 3 0.008559783
```

```
> plot(perf.srbct, col = color.mixo(5))
```

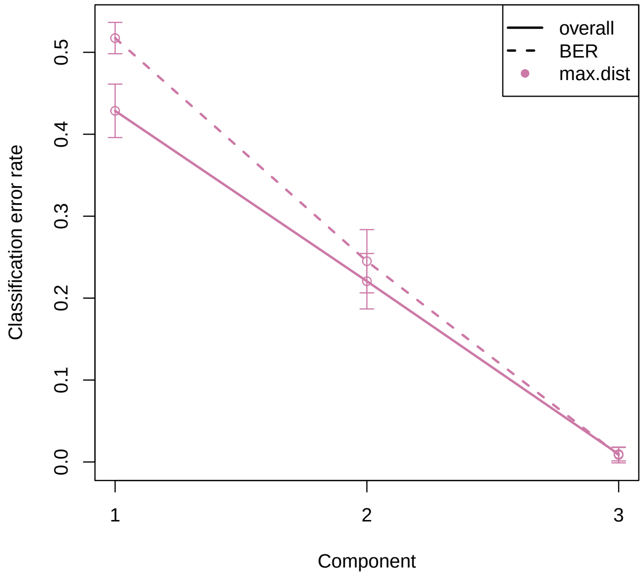

We can also examine the stability of the variables selected across the different cross-validation folds. Each variable's stability that is selected across the CV runs is represented with a vertical bar. We often observe a decrease in stability when more components are added in the model.

```
> par(mfrow=c(1,3))
> plot(perf.srbct$features$stable[[1]], type = 'h', ylab = 'Stability',
+      xlab = 'Features', main = 'Comp 1', las =2)
> plot(perf.srbct$features$stable[[2]], type = 'h', ylab = 'Stability',
+      xlab = 'Features', main = 'Comp 2', las =2)
> plot(perf.srbct$features$stable[[3]], type = 'h', ylab = 'Stability',
+      xlab = 'Features', main = 'Comp 3', las =2)
> par(mfrow=c(1,1))
```

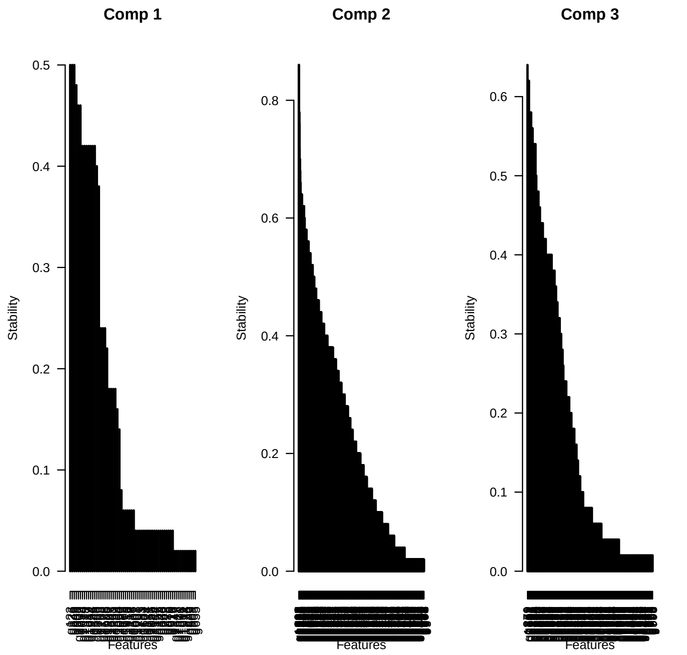

The selectVar function outputs the selected variables along with their loading weight value, from the most important to the least important variable. The code below also extracts the stability of the selected variables in order to gauge our confidence in the signature. For example on component 1, we observe a low stability of the selected variables:

```
> # here we match the selected variables to the stable features
> ind.match = match(selectVar(splsda.srbct, comp = 1)$name,
+                   names(perf.srbct$features$stable[[1]]))
> #extract the frequency of selection of those selected variables
> Freq = as.numeric(perf.srbct$features$stable[[1]][ind.match])
> data.frame(selectVar(splsda.srbct, comp = 1)$value, Freq)
```

```
         value.var Freq
g123  0.6491365782 0.50
g846  0.4482701086 0.50
g1606 0.3064356678 0.46
g335  0.3003358708 0.48
g836  0.2639666215 0.50
g783  0.2202765078 0.38
g758  0.2202614578 0.46
g1386 0.1625647718 0.40
g585  0.0207029259 0.24
g1158 0.0001252705 0.24
```

E1.5. Other graphical outputs

The plotLoading function displays the loading weights, where colors indicate the class for which the selected variable has a maximal mean value.

```
> plotLoadings(splsda.srbct, comp = 1, title = 'Loadings on comp 1',
+              contrib = 'max', method = 'mean')
```

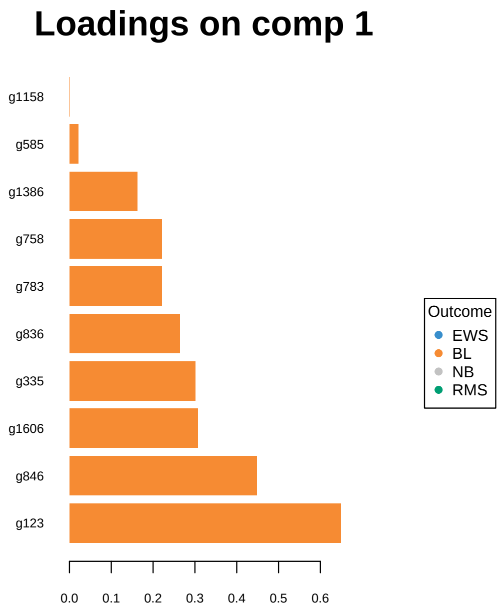


```
> plotLoadings(splsda.srbct, comp = 2, title = 'Loadings on comp 2',
+              contrib = 'max', method = 'mean')
```

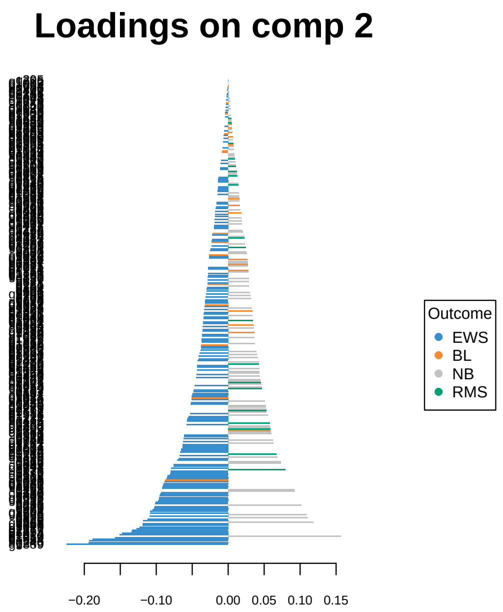

```
> plotLoadings(splsda.srbct, comp = 3, title = 'Loadings on comp 3',
+              contrib = 'max', method = 'mean')
```

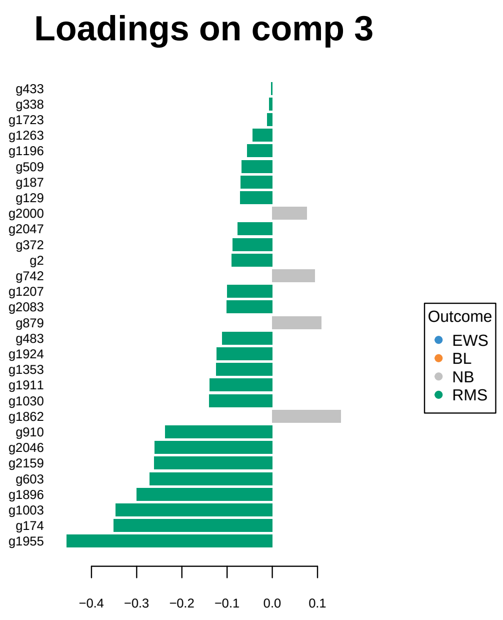

The color assigned can be changed to indicate the class with the minimal mean value:

```
> plotLoadings(splsda.srbct, comp = 1, title = 'Loadings on comp 1',
+              contrib = 'min', method = 'mean')
```

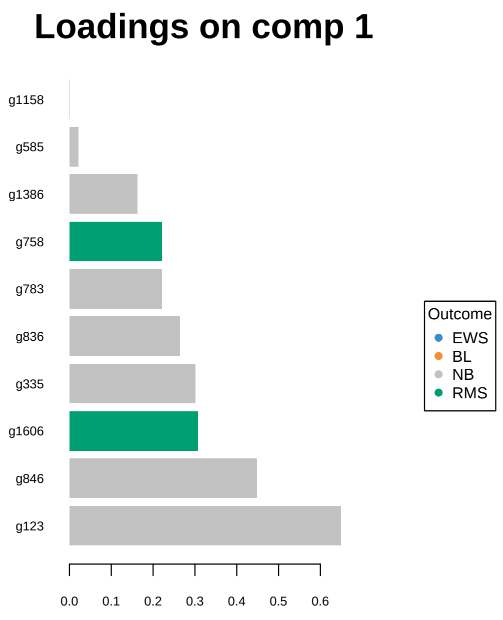

A Clustered Image Map including the final gene signature is plotted (default values to Euclidian distance and Complete linkage). The argument `comp` can be also be specified to highlight only the variables selected on specific components.

```
> cim(splsda.srbct)
```

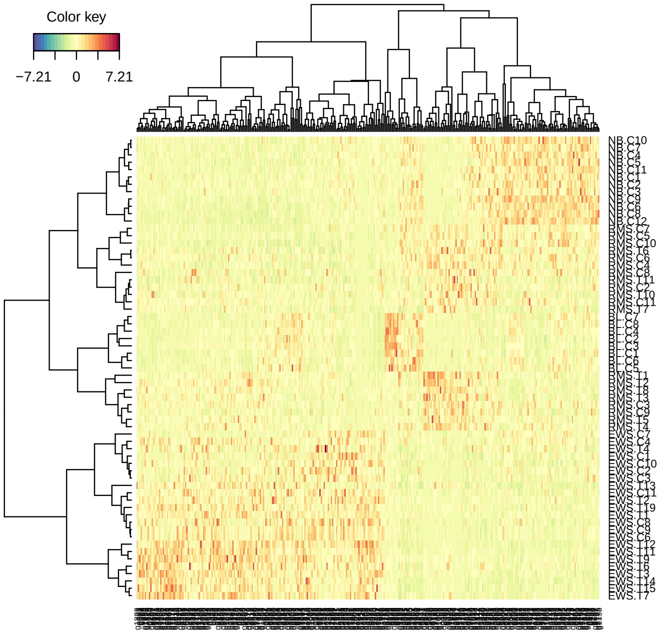


```
> cim(splsda.srbct, comp=1, title ="Component 1")
```

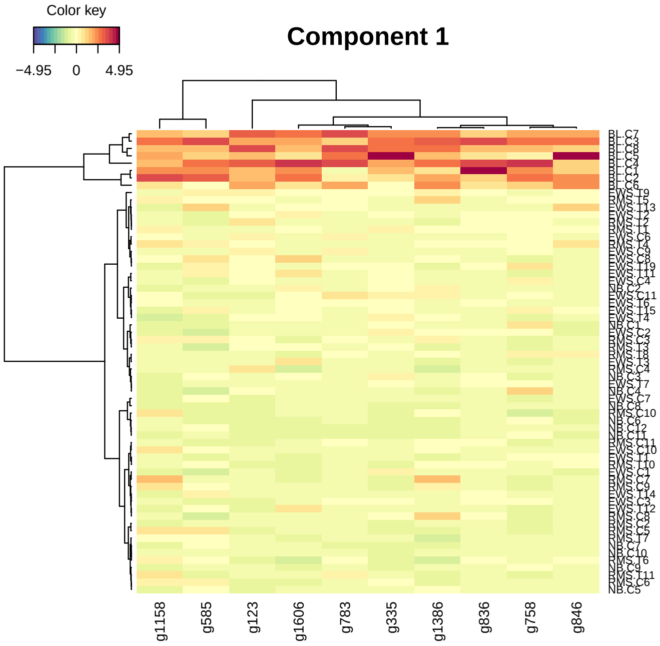

The arrow plot displays all samples spanned by the sPLS-DA components, with arrows pointing towards the location of the Y category (in the Y-space).

```
> plotArrow(splsda.srbct, legend=T)
```

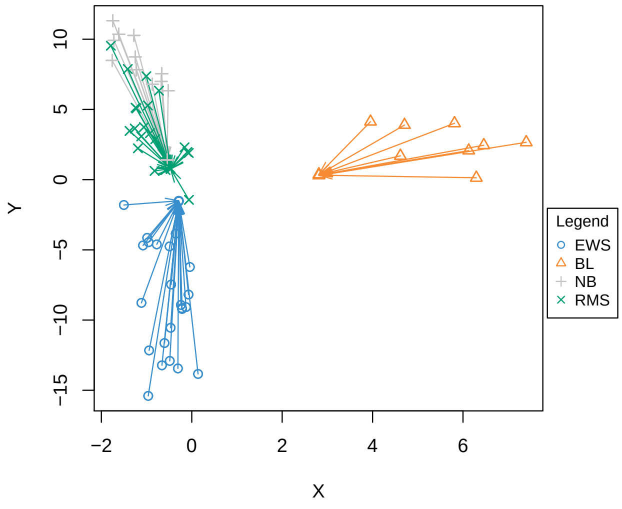

Other useful variable outputs include `plotVar` to display the selected variables on a correlation circle plot.

E1.6. Other outputs

If an external test set is available the `predict` function outputs the sPLS-DA predicted class membership for each test sample, see also an example from the Supplemental material regarding the prediction distances (Rohart et al., 2017).

E1.7. Session information of this Sweave code

```
> sessionInfo()
```

```
R version 3.4.1 (2017-06-30)
Platform: x86_64-apple-darwin15.6.0 (64-bit)
Running under: macOS Sierra 10.12.6

Matrix products: default
BLAS: /Library/Frameworks/R.framework/Versions/3.4/Resources/lib/libRblas.0.dylib
LAPACK: /Library/Frameworks/R.framework/Versions/3.4/Resources/lib/libRlapack.dylib

locale:
[1] en_AU.UTF-8/en_AU.UTF-8/en_AU.UTF-8/C/en_AU.UTF-8/en_AU.UTF-8

attached base packages:
[1] stats     graphics  grDevices utils     datasets  methods   base

other attached packages:
[1] mixOmics_6.3.0  ggplot2_2.2.1   lattice_0.20-35 MASS_7.3-47

loaded via a namespace (and not attached):
 [1] Rcpp_0.12.13       RSpectra_0.12-0    compiler_3.4.1     RColorBrewer_1.1-2
 [5] plyr_1.8.4         bindr_0.1          tools_3.4.1        digest_0.6.12
 [9] jsonlite_1.5       tibble_1.3.4       gtable_0.2.0       pkgconfig_2.0.1
[13] rlang_0.1.2        Matrix_1.2-11      igraph_1.1.2       shiny_1.0.5
[17] parallel_3.4.1     bindrcpp_0.2       gridExtra_2.3      stringr_1.2.0
[21] dplyr_0.7.4        knitr_1.17         htmlwidgets_0.9    grid_3.4.1
[25] glue_1.1.1         ellipse_0.3-8      R6_2.2.2           rARPACK_0.11-0
[29] rgl_0.98.1         tidyr_0.7.1        purrr_0.2.3        reshape2_1.4.2
[33] corpcor_1.6.9      magrittr_1.5       scales_0.5.0       htmltools_0.3.6
[37] matrixStats_0.52.2 assertthat_0.2.0   mime_0.5           colorspace_1.3-2
[41] xtable_1.8-2       httpuv_1.3.5       labeling_0.3       stringi_1.1.5
[45] lazyeval_0.2.0     munsell_0.4.3
```

```
Writing to file PLSDA-analysis.R
```

References

Javed Khan, Jun S Wei, Markus Ringner, Lao H Saal, Marc Ladanyi, Frank Westermann, Frank Berthold, Manfred Schwab, Cristina R Antonescu, Carsten Peterson, et al. Classification and diagnostic prediction of cancers using gene expression profiling and artificial neural networks. *Nature medicine*, 7(6):673–679, 2001.

Svante Wold, Michael Sjöström, and Lennart Eriksson. Pls-regression: a basic tool of chemometrics. *Chemometrics and intelligent laboratory systems*, 58(2):109–130, 2001.

Florian Rohart, Benoit Gautier, Amrit Singh, and Kim-Anh Lê Cao. mixomics: an r package for 'omics feature selection and multiple data integration. *bioRxiv*, 108597, 2017.
